# Structure, substrate binding and activity of a unique AAA+ protein: the BrxL phage restriction factor

**DOI:** 10.1093/nar/gkad083

**Published:** 2023-02-16

**Authors:** Betty W Shen, Lindsey A Doyle, Rachel Werther, Abigail A Westburg, Daniel P Bies, Stephanie I Walter, Yvette A Luyten, Richard D Morgan, Barry L Stoddard, Brett K Kaiser

**Affiliations:** Division of Basic Sciences, Fred Hutchinson Cancer Research Center, 1100 Fairview Ave. North, Seattle, WA 98109, USA; Division of Basic Sciences, Fred Hutchinson Cancer Research Center, 1100 Fairview Ave. North, Seattle, WA 98109, USA; Division of Basic Sciences, Fred Hutchinson Cancer Research Center, 1100 Fairview Ave. North, Seattle, WA 98109, USA; Department of Biology, Seattle University, 901 12th Avenue, Seattle, WA 98122, USA; Department of Biology, Seattle University, 901 12th Avenue, Seattle, WA 98122, USA; Department of Biology, Seattle University, 901 12th Avenue, Seattle, WA 98122, USA; New England Biolabs, 240 County Road, Ipswich, MA 01938, USA; New England Biolabs, 240 County Road, Ipswich, MA 01938, USA; Division of Basic Sciences, Fred Hutchinson Cancer Research Center, 1100 Fairview Ave. North, Seattle, WA 98109, USA; Department of Biology, Seattle University, 901 12th Avenue, Seattle, WA 98122, USA

## Abstract

Bacteriophage exclusion (‘BREX’) systems are multi-protein complexes encoded by a variety of bacteria and archaea that restrict phage by an unknown mechanism. One BREX factor, termed BrxL, has been noted to display sequence similarity to various AAA+ protein factors including Lon protease. In this study we describe multiple CryoEM structures of BrxL that demonstrate it to be a chambered, ATP-dependent DNA binding protein. The largest BrxL assemblage corresponds to a dimer of heptamers in the absence of bound DNA, versus a dimer of hexamers when DNA is bound in its central pore. The protein displays DNA-dependent ATPase activity, and ATP binding promotes assembly of the complex on DNA. Point mutations within several regions of the protein-DNA complex alter one or more *in vitro* behaviors and activities, including ATPase activity and ATP-dependent association with DNA. However, only the disruption of the ATPase active site fully eliminates phage restriction, indicating that other mutations can still complement BrxL function within the context of an otherwise intact BREX system. BrxL displays significant structural homology to MCM subunits (the replicative helicase in archaea and eukaryotes), implying that it and other BREX factors may collaborate to disrupt initiation of phage DNA replication.

## INTRODUCTION

The ancient and ongoing battle between single cell microbes and phage has led to the evolution of a wide variety of phage restriction systems, including those that span innate and adaptive mechanisms for surveillance and degradation of foreign genomes (1–5). Originally discovered in the early 1980s (6), phage growth limitation (‘Pgl’ (7)) or bacte**r**iophage exclusion (‘BREX’ (8,9)) systems are found in most bacterial and archaeal species, where they participate in defense against phage invasion through poorly understood mechanism(s) (9). BREX systems are encoded by single operons within genetic defense islands, and are currently categorized into six subtypes based on the number of genes in each system (typically between four and eight) and sequence-based functional annotation of proteins encoded by those genes (9).

All known BREX systems have two genes in common. The first contains an alkaline phosphatase domain and is named ‘*PglZ*’; the second contains an ATPase domain and is alternatively referred to as ‘*PglY*’ or ‘*BrxC*’ (9). Similar to classic restriction-modification (‘RM’) systems, the majority of BREX systems also contain a site-specific methyltransferase (‘PglX’) that modifies the host genome and presumably protects it from the action of the BREX system (9,10). At least one alternative BREX system instead encodes a PAPS reductase gene that also modifies host DNA (11). However, in contrast to RM systems, BREX systems do not appear to encode a nuclease and do not induce nucleolytic degradation of the phage genome (7,9,12,13). In addition, the deletion or inactivation of the methyltransferase in the presence of an otherwise intact BREX system is not toxic, and host methylation requires several additional members of the BREX family (9,12,14). Of the various BREX protein factors, a detailed functional and structural analysis has only been described only for the BrxR regulatory protein, which is found in a subset of type I BREX systems (14,15). More recently, crystal structures of BrxA have also been described (16,17), however a specific role for that protein in BREX function has not yet been described.

Two separate studies recently described type I BREX systems from *Acinetobacter sp. 394* and from *Escherichiafergusonii* (14,15). Similar to other previously described type I BREX systems (9,12), the protein factors encoded within each of those bacterial operons contain protein sequences with recognizable homology to DNA binding domains (BrxA), DNA methyltransferase domains (PglX), phosphatase domains (PglZ), ATPase domains (BrxC and BrxL) and origin replication complex (ORC) subunits (BrxB and BrxC). Several of the BREX subunits are quite large (over 1000 residues) and a substantial fraction of their amino acid sequences do not correspond to a clearly predicted protein fold. An analysis of the effect of precise deletions of each gene in the *Acinetobacter* sp. 394 system (14) indicated that all of the genes in that system are required for significant phage restriction function (whereas only five of the seven genes are required for host chromosomal methylation).

The final gene in most type 1 BREX systems, termed ‘BrxL’, encodes a large protein (670–700 residues in various species), with much of its sequence predicted to adopt a fold corresponding to the chambered ATP-dependent AAA+ protein family, which are involved in a wide variety of cellular functions throughout all biological kingdoms (18). Such proteins include protease enzymes (such as LonP) (19) and helicases (such as RadA) (20). The sequence similarities between BrxL and either Lon or RadA are comparable (both less than 20% identity versus BrxL) and is highest across recognizable sequence motifs required for nucleotide binding and ATPase activity. Although BrxL is often annotated in various sequence databases as a homologue of Lon protease (a designation that led to its name), it does not contain an obviously conserved Lon protease active site motif (21). The BrxL gene is limited to only Type 1 and 4 BREX systems; in alternative BREX systems (four total) it appears to have been replaced by a DNA helicase (9). Furthermore, a recent study of a variant BREX system in *Salmonella*, termed the *stySA* RM system, showed that BrxL is required for restriction only, and concludes that it is unlikely to be a AAA+ protease (21).

In this study, we describe a combination of CryoEM structure determinations, *in vitro* biochemical analyses, and *in cellulo* phage restriction assays that demonstrate that BrxL is a novel AAA+ protein factor, possesses ATPase activity and displays ATP-dependent assembly on DNA, and that disruption of its ATPase active site results in the same reduction in BREX phage restriction as the deletion of the entire BrxL gene from the operon. Beyond the immediate characterization of the structure and function of the BrxL protein, the study also provides a detailed view of the conversion of such a protein factor from an unbound heptameric to substrate-bound hexameric assembly, and one of the first structures of a AAA+ protein trapped in an extended complex with its biomolecular substrate.

## MATERIALS AND METHODS

Materials and methods employed for the identification and subcloning of the intact type I BREX system from *Acinetobacter* 394, for subsequent manipulations to generate validated constructs harboring precise deletions or mutations of individual BREX factor coding sequences (including BrxL) within that system, and for bacterial growth and phage restriction assays were previously described in detail (14) and performed identically in this study. Detailed description of those methods are duplicated in full from the text of (14) in the Supplementary Methods provided for this current study.

### Protein expression and purification

The BrxL gene was subcloned from the native *Acinetobacter* sp. 394 locus into a commercially available pET15b plasmid (Novagen, Inc) that incorporates an N-terminal, thrombin-cleavable 6XHistidine (His6) tag fused to the protein of interest. Inductions were carried out in BL21 (DE3) pLysS *E*scherichia *coli* cells. For inductions, a single colony from an LB-ampicillin plate was introduced into a 10 ml tube of LB media containing ampicillin (100 μg/ml) and grown at 37°C overnight. The following morning, that culture was diluted 100-fold into 1 liter of the same media and incubated at 37°C with shaking until an OD_600_ of 0.6 was reached. After 20 min cooling in a 4°C cold room, IPTG was added to a final concentration of 500 μM, and the culture was shaken for an additional 20–24 h at 16°C. Cell pellets were then harvested by centrifugation and stored at −20°C.

For purification of His-tagged protein generated using the pET15b expression vector, cell pellets were resuspended in lysis buffer (25 mM Tris (pH 7.5), 500 mM NaCl, 20 mM imidazole), lysed by sonication on ice and centrifuged in an SS34 rotor for 25 min at 18 000 rpm, and the supernatant was filtered through a 5 μm syringe filter. The clarified lysate was incubated in batch with Ni-NTA agarose resin (Qiagen) for 1 h at 4°C with rotation. The resin was transferred to a gravity filtration column (Bio-Rad), washed with >50 volumes of lysis buffer at 4°C and eluted in lysis buffer supplemented with 200 mM imidazole.

The sample was concentrated to a volume of 1–2 ml, filtered through a 0.22 μm centrifugal filter and loaded onto a HiLoad 16/60 Superdex 200 prep grade size exclusion column (Millipore Sigma) equilibrated in 25 mM Tris (pH 7.5),150 mM NaCl, 10 mM MgCl_2_. Peak fractions were pooled and concentrated using an Amicon spin filter with a 30 kDa molecular mass cutoff to a final concentration of ∼14 mg/ml. Aliquots were stored long-term at –20°C in 50% glycerol v/v.

### Mutagenesis of BrxL

For all cloning manipulations, correct sequences were verified by whole plasmid sequencing (Primordium Inc). Individual point mutations were introduced into the BrxL.pET15b expression construct with the Q5® mutagenesis kit (New England Biolabs) using the manufacturer's provided protocol. The pET15b constructs were then used to subclone BrxL mutants into the intact BREX operon of the pACYC vector using a 3-part Gibson assembly strategy. The backbone of pACYC-BREX lacking the BrxL coding sequence was amplified using Q5 polymerase (New England Biolabs) in two separate PCR reactions, both using pACYC-BREX_WT_ as the template and primer pairs DB5/DB9 and DB6/DB10 (Supplementary Table S1). The full coding sequence of BrxL containing various point mutations was amplified from pET15b plasmids described above using primer pair DB7/DB8. The three PCR amplicons were gel purified (Zymo Research Zymoclean Large Fragment kit, catalog #11–301L) and assembled into an intact plasmid using 2× HiFi Assembly mix (New England Biolabs). Reactions were transformed into *E. coli* strain ER2683 (competent cells kindly provided by New England Biolabs) and selected on LB/agar plates containing 25 μg/ml chloramphenicol. Initial attempts to obtain BrxL mutants in pACYC-BREX by transforming Gibson assembly reactions into DH10B competent *E. coli* invariably produced inactivating mutations in some part of the BREX operon.

### Crystallographic structure determination of the BrxL C-terminal domain

Limited proteolysis experiments of full-length BrxL indicated proteolytic sensitivity near residue 500, resulting in the production of stable protein domains flanking that location. Subsequently, the final 200 residues of the protein (corresponding to amino acids 498–697) were subcloned into the expression vectors described above, and the corresponding region of the protein was purified and crystallized. The crystals were grown by hanging drop vapor diffusion in drops set with 1 μl of protein (protein concentration approximately 5 mg/ml) plus 1 μl of well solution (8% w/v PEG8000, 200 mM KCl, 0.1 M HEPES 7.4) and found to belong to space group F32, with four copies of the protein domain per asymmetric unit. For cryopreservation, crystals were transferred into crystallization solution containing 20% ethylene glycol, incubated for one minute and flash frozen in liquid nitrogen. Diffraction data were collected at the Advanced Light Source synchrotron facility (ALS, Berkeley, CA) at beamline 5.0.1. The structure of the protein domain was determined to 2.25 Å resolution via molecular replacement, using a structural model produced by the AlphaFold structure prediction server (22) as a phasing search model.

Data were indexed and scaled using HKL2000 software (23). Subsequent rounds of building were carried out in Coot (24) and refinement was performed using Refmac5 (25). See Table 1 for data collection and refinement statistics.

**Table 1.** Crystallographic data collection and refinement statistics

PDB ID	8EIL
**Data Collection**	
Space group	F23
Unit cell	
*a*, *b*, *c* (Å)	213.34, 213.34, 213.34
alpha, beta, gamma (°)	90, 90, 90
Wavelength (Å)	0.9774
Resolution range (Å)	50.0–2.25 (2.29–2.25)
Unique reflections	37992
*R*-merge	0.159 (2.636)
*R*-meas	0.161 (2.679)
*R*-pim	0.025 (0.476)
CC1/2	(0.581)
*I*/sigma(*I*)	25.0 (1.0)
Chi^2^	0.536
Multiplicity	29.8 (31.3)
Completeness (%)	100.0 (100.0)
Wilson *B*-factor	45.3
**Refinement**	
*R*-work	0.2162
*R*-free	0.2306
Number of non-hydrogen atoms	5208
macromolecules	5066
ligands	22
water	120
Protein residues	716
RMS(bonds)	0.003
RMS(angles)	0.67
Ramachandran favored (%)	98.28
Ramachandran allowed (%)	1.58
Ramachandran outliers (%)	0.14
Clashscore	5.48
Average *B*-factor	61.66
macromolecules	61.93
ligands	66.37
solvent	49.38

### CryoEM structure determination of full length unbound and DNA-bound BrxL

A schematic of the methods described below are shown in Supplementary Figure S1 and a summary of data processing and subsequent modeling is shown in Table 2. Negative-stain grids were prepared by the application of 4 μl of SEC purified BrxL samples to a glow-discharged uniform carbon film coated 400 mesh copper grid. The particles were allowed to adsorb to the film for 60 seconds. Excess solution was wicked away by briefly touching the edge of a filter paper. The grid was quickly washed three times with 20 μl drops of water and once with 20 μl of 0.5% uranyl formate (UF) followed by staining for 20 seconds with another 40 μl drop of UF. The grids were air-dried for at least 2 h prior to inspection on an in-house JEOL JM1400 microscope (operating at 120 kV) equipped with a GATAN Rio 4kx4k CMOS detector. Both BrxL(WT) and BrxL(E280Q) distributed homogeneously in random orientations over the surface of the carbon film in negative stained preparations. Screening for vitrification conditions and initial data collection was performed using a GLACIOS 200 kV electron microscope equipped with a GATAN K2 direct electron detector.

**Table 2.** CryoEM data collection, refinement, and validation statistics

Data collection	Wild-type BrxL	E280Q-BrxL
EM equipment	Glacios	Krios
Voltage (kV)	200	300
Detector	Gatan K3	Gatan K3
Pixel size (Å/Pixel)	0.561	0.5347
Electron dose (e^−^/Å^2^)	50	50
Defocus range (μm)	0.1–4.0	0.1–4.0
Micrographs collected	2982	7430
Micrographs used	2906	5298
Reconstruction:		
Software	cryoSPARC	cryoSPARC
Number of used particles	22 162	234 715
Symmetry	C7	No symmetry
Resolution (Å)	3.55	3.62
Map sharpening *B*-factor (Å^2^)	74.5	96.8
Refinement:		
PDB/EMD ID	8EMC/EMD-28244	8EMH/EMD-28248
Software	Phenix	Phenix
Cell dimensions		
*a* = *b* = *c* (Å)	454.72	384.98
$\;{\rm{\alpha }} = {\rm{ \beta }} = \gamma ( \circ )$	90	90
Model composition:		
Protein residues	9328	8025
Nucleotide	—-	127
Side chains assigned	9328	8025
MolProbity score:	1.97	1.79
Rms deviations:		
Bonds length (Å)	0.004	0.004
Bonds angle (°)	0.731	0.687
Ramachandran plot statistics (%):		
Favored	95.01	97.08
Allowed	4.70	2.81
Outlier	0.11	0.29

#### Wild-type BrxL data collection and processing

CryoEM grids were prepared by applying 2.5 μl of purified BrxL at ∼0.8 mg/ml (OD260/OD280 ratio ∼0.56) to a glow-discharged Quantifoil1.2/1.3 holey carbon film coated copper grid, which was blotted for 7.0 s at a Tension of 10, and plunge-frozen in liquid ethane using an FEI Vitrobot Mark IV. Screening datasets with a total of 100 movies were collected on a GLACIOS electron-microscope (operating at 200 kV) equipped with a GATAN K2-Summit direct electron detector at a pixel size of 1.16 Å. Most images showed extensive aggregation of an oval basket with a few free-standing particles. A preliminary dataset comprising approximately 100 movies was collected from this highly aggregated specimen. 514 particles were manually picked and extracted with a box size of 300 pixel. All 514 particles were categorized into nine unique classes upon 2D classification requesting for 10 classes. *Ab-initio* 3D reconstruction yielded a map with rough 7-fold symmetry. Homogenous refinement with C7 symmetry resulted in a cage-like particle suggestive of a dimer of heptamers.

A larger dataset of ∼3000 movies was then collected at a pixel size of 1.12 Å (super res 0.56 Å/pixel) using an in-house FEI GLACIOS microscope equipped with a FALCON DE-10 direct elector detector. The same 2D template from the initial dataset was used as template for template picking. After two rounds of 2D classification and selection, 111, 411 particles were reextracted with a box size of 406 pixels and Fourier cropped to a box size of 392 pixels in order to be combined with 6,722 particles extracted from template-picking and 2D-classification of the initial small dataset. Three-dimensional (3D) reconstruction and local refinement with C7 symmetry resulted in a map with a Gold Standard Fourier Shell Correlation (‘GSFSC’) of 3.54 Å at a FSC of 0.143 between the two half maps.

#### BrxL-E280Q data collection and processing

Samples of BrxL (E280Q) eluted from the SEC column display an approximate four-fold higher OD_260_/OD_280_ ratio than BrxL (WT) protein, suggesting co-elution with DNA fragments during extraction and purification of the samples. CryoEM screening showed abundant tadpole-like particles with occasional ‘beads-on-a-string’ images. A full dataset with 7000 movies was collected at the Pacific North CryoEM Center (PNCC) at a pixel size of 1.0694 Å (0.5347 Å superres) using a FEI KRIOS microscope (operating at 300 KV) equipped with a K3-summet Direct Electron detector. Blotpicker (120 Å and 220 Å were used as minimum and maximum diameters respectively) was used to pick particles from 1000 movies. After 2D classification/selection, 16 template images were used for template picking from 5298 accepted movies. 3.4 million particles were extracted at a box size of 400 pixel and rendered two rounds of 2D classification/selection.

*Ab-initio* 3D reconstruction of selected particles with four initial models revealed two different types of full particles (50% and 24% respectively), half particles (14%) and fragments (11%). Unexpectedly, homogenous refinement with C1 symmetry revealed 6-fold instead of 7-fold symmetry and clear densities for double strand DNA through the center of the protein complex. Because of the absence of 6-fold symmetry in the DNA double strand, subsequent refinements were all carried out in C1 symmetry leading to a map with GSFSC of 3.63 Å at a FSC of 0.143 between the two half maps.

For both structures, all data preprocessing, which includes motion correction, contrast transfer function (CTF) estimation, and exposure curation, as well as 2D particle selection, 3D-model reconstruction/refinement, and post refinement were performed using the software package cryoSPARC2 (26). For each movie stack, the frames were aligned for beam-induced motion correction using Patch-motion-correction. Patch-CTF was used to determine the CTF parameters. Bad movies were eliminated based on a CTF-fit resolution cutoff at 5 Å and relative ice thickness of 1.2 estimated from the CTF function by cryoSPARC2. Different particle picking algorithms, including manual, template-based and blob picking were employed to the same dataset and results on model distribution were compared. The evaluation of the density map at all stages (and comparison with the AlphaFold predicted model) to the final density map were accomplished in Chimera (27). The final structures were built and refined with program COOT (24).

Refined maps of the wild-type and E280Q mutant of BrxL at ∼3.6 Å showed excellent details of the secondary structure of the peptides, and in the latter case, densities of the double strand DNA phosphodiester back bone. We were able to build with high certainty the Cα-back bone more than 400 out of 679 residues, which clearly displayed the typical fold of an AAA+ protein. A superposition with a model generated using AlphaFold not only confirmed the tracing of our initial model but also provided the register of residues in the AAA+ domain. Once the registration was confirmed, final manual fitting of all 679 residues and their sidechains into the density were carried out in COOT (24) with subroutines Sphere and Tendon.

### DNA binding assays

Association of BrxL and various point mutants with DNA was determined using electrophoretic mobility shift assays (EMSA). Purified BrxL proteins (ranging in concentration from 0.2 to 2 μM) were incubated with a 207 basepair dsDNA template (corresponding to the region surrounding the BrxR promoter; see Supplementary Table S1). The dsDNA template was generated by PCR using Q5 polymerase (NEB), purified using a Clean and Concentrate kit (Zymo research) and used at a final concentration of 20 nM in binding assays. The ssDNA template was synthesized as a 100 nucleotide fragment (Integrated DNA Technologies; see Supplementary Table S1). Binding reactions were incubated in a 20 mM Tris, pH 7.9, 40 mM NaCl, 5 mM MgCl_2_ and 2.5% sucrose for 45 min at 23°C. Samples were separated on a 6.5% acrylamide gel poured in 0.5× TBE, using 1× TBE as a running buffer. Gels were pre-ran for 30 min, 90 V prior to loading samples and the wells were flushed with 1× TBE using a syringe. The acrylamide stock used to pour the gels was a 40% solution of 29:1 acrylamide:bis-acrylamide (BioRad). 4 μl of binding reactions were loaded onto the gels and electrophoresed for 70 min at 90 V. The gels were stained in 1X SYBRGold (made up in 1× TBE) for 30 min and visualized on a BioRad Gel Doc Imaging system.

### ATPase assays

Assays were performed using Abcam's ATPase Colorimetric Assay Kit (Cat. No. ab234055) following the provided protocol in a 96-well plate (Porvair 96-Well Polystyrene, Clear, Flat Bottom, 350 µl Assay Plate; Thomas Scientific, Cat. No. 1167V76). Phosphate standards containing 0–5 nmol phosphate per well were prepared in triplicate in the kit-provided ATPase Assay Buffer. Monomeric BrxL protein constructs (wild-type and various mutants) were used at a final concentration of 1500 nM. Activity was tested in the absence of nucleic acids or in the presence of 3, 15 or 30 nM dsDNA. The dsDNA used in ATPase assays is the same 207 bp DNA fragment described in EMSA assays. Reactions were run in duplicate in a final volume of 100 μl with ATPase Assay Buffer. Control reactions (run in triplicate) included the ATPase Assay Buffer only (‘Reagent Control’) and wells containing 2, 10 or 20 microliters of the provided Positive Control reagent. To initiate the reactions, 100 μl of Reaction Master Mix (2 μl of ATPase Substrate plus 98 μl ATPase Assay Buffer per reaction) was added to each reaction, excluding the Phosphate Standards, and incubated for 30 min at 23°C. 30 μl of ATPase Assay Developer was then added to all wells and incubated for another 30 min at room temperature. The absorbance of all wells was read at 650 nm on a SpectraMax M5 plate reader (Molecular Devices). The 0 nmol Phosphate Standard average reading was subtracted from the remaining standards and a Phosphate Standard curve was plotted. The average Reagent Control reading was subtracted from the average of the sample wells and the corrected sample readings were applied to the phosphate standard curve, using the slope-intercept formula, to determine the nmol of phosphate generated during the reaction time. The ATPase Activity per nmol of BrxL was determined using the following equation:


$$\begin{equation*}\frac{B}{{\left( {t*c} \right)}}\, = \,\frac{{nmol\,phosphate}}{{{\rm{min}}\,*nmol\,BrxL}}\end{equation*}$$

were *B* = nmol of phosphate generated, *t* = reaction time in min and *c* = nmol of BrxL.

### Size exclusion chromatography to measure BrxL multimeric assembly

BrxL constructs (WT and E280Q) were purified via metal affinity chromatography and SEC, and fractions from the monomer peak were flash frozen and stored at -80°C in the presence of 20% glycerol. To assess if ATP or AMP-PNP induced multimer assembly in the absence of DNA, a sample was thawed on ice, diluted with 20 mM Tris (pH 7.5), 150 mM NaCl, 5 mM MgCl_2_ and concentrated to ∼2.8 mg/ml (∼39 μM); the final concentration of glycerol was ∼5%. The sample was split into three aliquots and AMP-PNP (Roche) or ATP (Cytiva) were added to a final concentration of 1.25 mM; a third sample had no added nucleotide. Reactions were incubated at room temperature for 60 min, then loaded onto a HiLoad 16/60 Supderdex 200 (Cytiva) equilibrated in 25 mM Tris (pH 7.5), 150 mM NaCl and eluted over 130 ml at 1.5 ml min^−1^.

To assess multimer assembly in the presence of DNA, 20 μM BrxL (WT or E280Q) was incubated with 0.25 μM dsDNA (the same 207 bp fragment used for EMSA assays) in a final volume of 200 μl under the same conditions described above. Samples were then separated on an SEC650 analytical column (BioRad). Where indicated, ATP and AMP-PNP were used at 1.25 mM final concentrations.

### Phage restriction assays

As described previously (14) and repeated in detail in *Supplementary Methods*, phage restriction activity was evaluated by determining the fold change in phage plaque formation using λ_vir_ phage and *E. coli* strain ER2683 as a function of the presence, absence or mutation of the BrxL gene in the BREX operon. Log phase cultures of cells transformed with the indicated pACYC constructs were mixed with 0.5% top agar, plated on chloramphenicol plates (25 μg/ml) to form a lawn, then spotted with 10-fold serial dilutions of λ_vir_ phage. For each construct tested, we performed a minimum of three (and up to eight) biological replicate experiments, each of which corresponded to two separate technical replicates.

## RESULTS

### Crystal structure of the BrxL C-terminal domain

**Figure 1.**
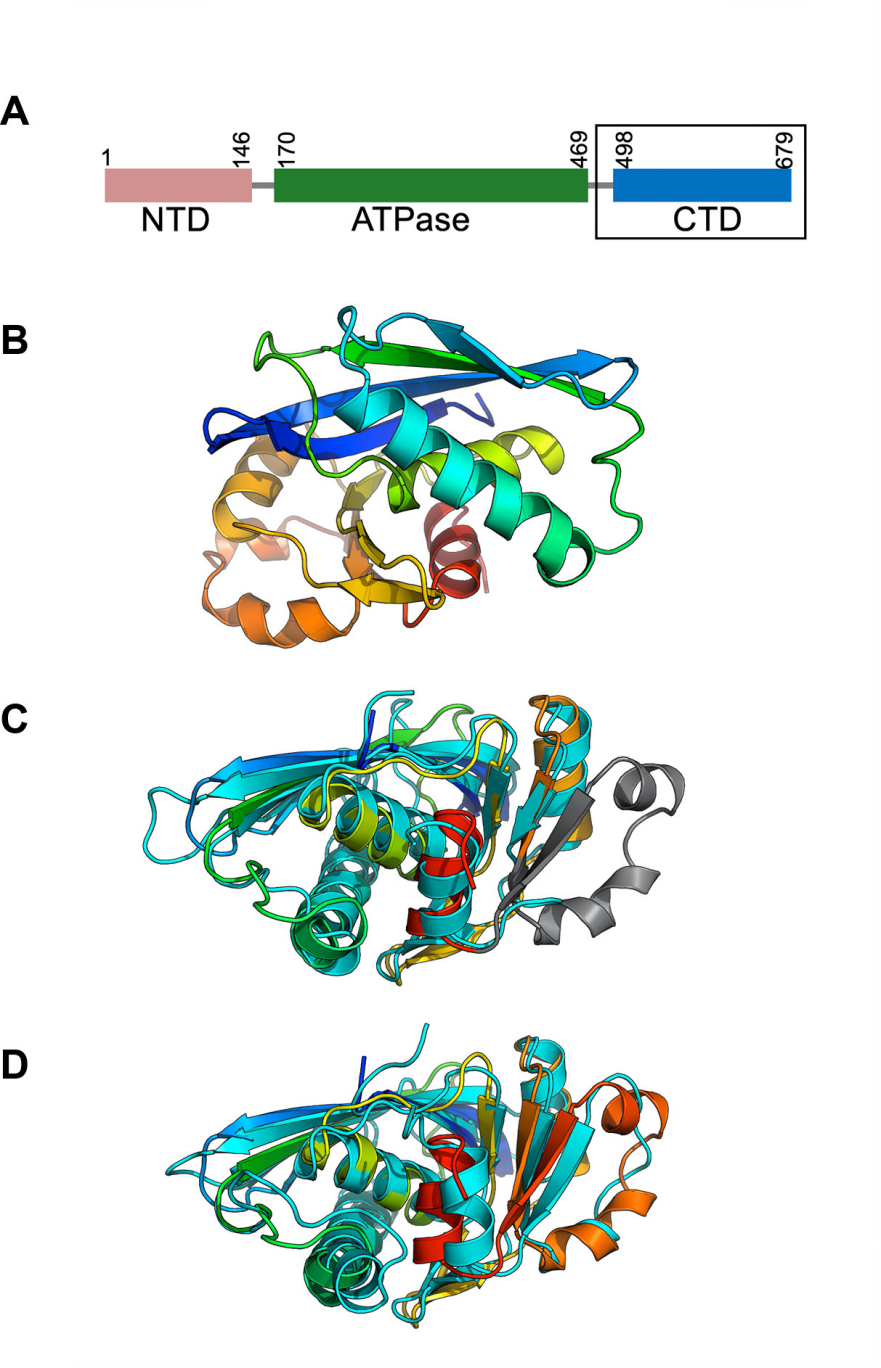
Domain organization of BrxL and crystal structure of its C-terminal domain. Panel **A**: BrxL is predicted to contain an N-terminal domain (‘NTD’) with no obvious sequence homology to previously described protein domains, a central AAA+ ATPase domain, and a C-terminal domain (‘CTD’; boxed) that displays low sequence identity (∼14%) to the same region in either Lon protease (LonP) or RadA. Panel **B**: Ribbon diagram of crystallographic model of BrxL_C_ (boxed in panel A) colored in a spectrum from blue (N-terminus) to red (C-terminus). Panels **C** and **D**: superposition of the BrxL_C_ structure to its two closest structural homologues in the RCSB database: the C-terminal domain of Lon protease (‘LonP_C_’) from *Methanocaldococcus jannaschii* (PDB 1XHK; panel C) (27) and the C-terminal domain of RadA (‘RadA_C_’) from *Thermus thermophilus* (PDB 5H45; panel D) (19). See also Supplementary Figure S1.

BrxL is predicted to contain three domains (Figure 1A): an N-terminal domain (approximately residues 1–146) with no obvious sequence homology to previously described protein domains, a central domain (approximately residues 170–469) with obvious homology to AAA+ ATPase proteins, and a C-terminal domain (approximately residues 498–679; here termed ‘BrxL_C_’) that displays low sequence identity (14%) to the same region in either Lon protease (‘LonP_C_’) or RadA (‘RadA_C_’).

To better understand how BrxL’s C-terminal domain relates to those or other protein domains (and to provide a starting model of that domain for subsequent CryoEM studies of the full-length protein) we expressed, purified, and crystallized BrxL_C_. During the final step of purification by size exclusion chromatography (SEC), we observed that BrxL_C_ eluted at a retention volume consistent with a monomer (Supplementary Figure S2a) rather than as a higher order multimers (eventually shown to correspond to hexamers) previously observed in studies of LonP_C_ and RadA_C_. We obtained crystals and solved the X-ray structure to 2.25 Å resolution (Table 1, Figure 1B). The crystal structure contains 4 subunits in the asymmetric unit, with no obvious higher order symmetry.

The two closest structural homologs of BrxL_C_, identified by the DALI server (28) are LonP_C_ from *Methanocaldococcus jannaschii* (PDB 1XHK) (29) and RadA_C_ from *Thermus thermophilus* (PDB 5H45) (20) (Figure 1C and D). LonP functions as a protease that fully unfolds and threads misfolded polypeptides through its central pore (resulting in their proteolytic degradation by its C-terminal protease domain) whereas RadA is a DNA helicase. The backbone atoms of BrxL_C_ superpose well with both domains despite low amino acid sequence identity (BrxL_C_ shares 14% identity with both domains); superposition of BrxL_C_ with Rad_C_ generates an RMSD value for 148 structurally equivalent α-carbons of 2.3 Å (*P*-value 8.97 × 10^−14^); superposition with LonP_C_ generates an RMSD value for 172 structurally equivalent α-carbons of 3.3 Å (*P*-value 3.02 × 10^−12^).

A structure-based sequence alignment of the BrxL_C_ with RadA_C_ or LonP_C_ does not demonstrate obvious conservation with previously visualized DNA binding residues with RadA_C_ or with a protease catalytic motif as observed in LonP_C_ (Supplementary Figure S2b). BrxL_C_’s distinct solution and assembly behaviors, in conjunction with the low sequence conservation relative to LonP_C_ and RadA_C_, indicate that the BrxL_C_ domain may have adopted a distinct structural role in the function of BrxL as compared to the same domain in LonP or RadA.

### CryoEM analysis of BrxL

To study the structure of full-length BrxL we next purified two constructs, wild-type BrxL (BrxL_WT_) and a variant containing a catalytic point mutation in the ATPase ‘Walker B’ catalytic motif (BrxL_E280Q_). When we initiated this study we hypothesized that BrxL may encode a Lon protease homologue and anticipated using the E280Q mutant as a ‘substrate trap’ to identify potential polypeptide substrates, as has been used for other Lon homologs (30). As well, an analogous mutation in the murine AAA+ protein TRIP13 significantly increased the yield of protein multimers during its purification (31).

The constructs were each expressed in *E. coli* and purified by metal affinity chromatography followed by size exclusion chromatography (SEC) (Supplementary Figure S3). Both constructs eluted from SEC as a mixture of monomers and one or more higher order complexes, with the E280Q variant displaying a substantial increase in higher order multimeric peaks and an ∼4-fold higher OD_260/280_ ratio than the wild-type BrxL construct (indicating an increase in co-purifying nucleic acids). Individual fractions corresponding to various elution volumes for both constructs were subsequently used when screening grids for CryoEM analyses in the absence of added ATP; ultimately grids generated from fractions corresponding to high molecular weight species were used for structural analyses.

**Figure 2.**
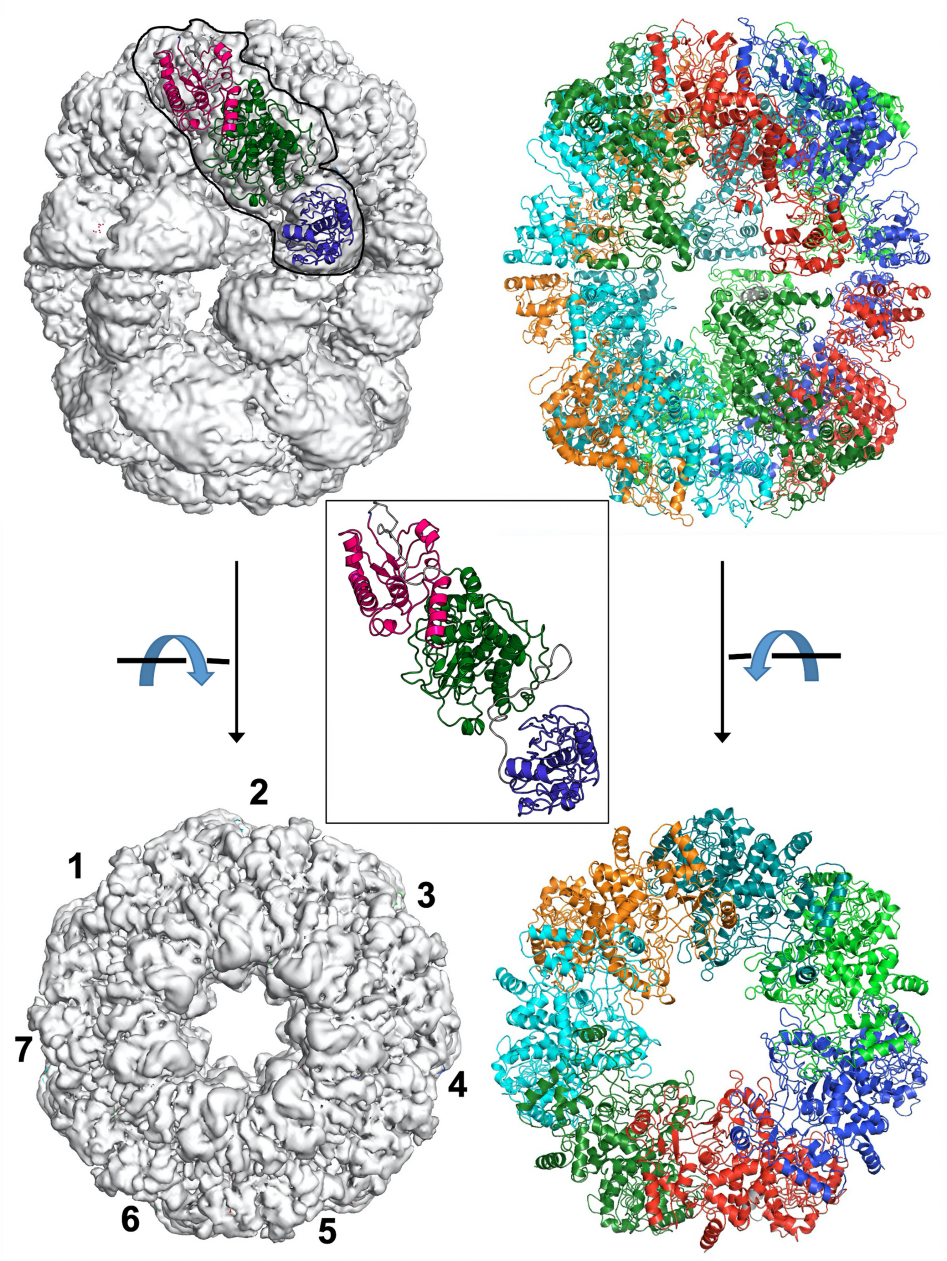
CryoEM analysis of wild-type unbound BrxL. Left panels: CryoEM map (shown looking across the axis of the protein barrel and down the axis of the barrel, respectively) corresponding to approximately 3.5 Å resolution. See also Supplementary Figure S2a. The map displays seven-fold symmetry accompanied by an orthogonal dyad symmetry axis, corresponding to 14 subunits arranged around a large, unoccupied central channel. A ribbon diagram of a single BrxL subunit (inset and also superimposed on electron density), with domains individually colored as in Figure 1a is superimposed and outlined against the electron density of one corresponding subunit. Right: panels: Corresponding ribbon diagram of the DNA-free BrxL assemblage (containing 14 individual subunits) in the same orientation. The protein assemblage corresponds to a tail-to-tail dimer of heptamers, with contacts around the two ‘poles’ between the NTD and ATPase domains driving heptamer formation and additional contacts across the ‘equator’ between CTDs driving dimerization of the heptamers.

EM grids of wild-type BrxL contained a mixture of particle sizes, with the majority appearing to comprise barrel-shaped multimers displaying seven-fold (heptameric) symmetry. Smaller particles observed on the grids appear to correspond to dissociated half-cylinders (containing a single ring of seven subunits) and smaller protein sub-complexes. The final CryoEM maps for the full size wild-type BrxL complex extended to approximately 3.5 Å resolution. The structure of the full-length BrxL assemblage was built and refined into that density using homology models of the NTD and AAA+ ATPase domains generated using AlphaFold (32) combined with the crystal structure of the C-terminal domain described above. Ultimately the maps supported modeling 14 independently placed BrxL subunits arranged into a dimer of heptameric rings (Figure 2 and Supplementary Figure S1a).

In the resulting structure, each heptameric ring assembles via contacts between the NTD and central ATPase domains of adjacent monomers. Two such heptamers further associate with one another via tail-to-tail contacts between their CTDs. A large internal void in the interior of the protein chamber contains no significant electron density. The buried surface area between adjacent protein subunits around each heptameric ring (as calculated using the PISA webserver (33)) is approximately 1100 Å^2^ and involves up to six directional side chain hydrogen bonds and salt bridges distributed between eleven residues within each interface. The buried surface area between each pair of CTDs around the equator of the assemblage (producing the tail-to-tail dimerization of two heptameric rings) is smaller (∼600 Å^2^).

**Figure 3.**
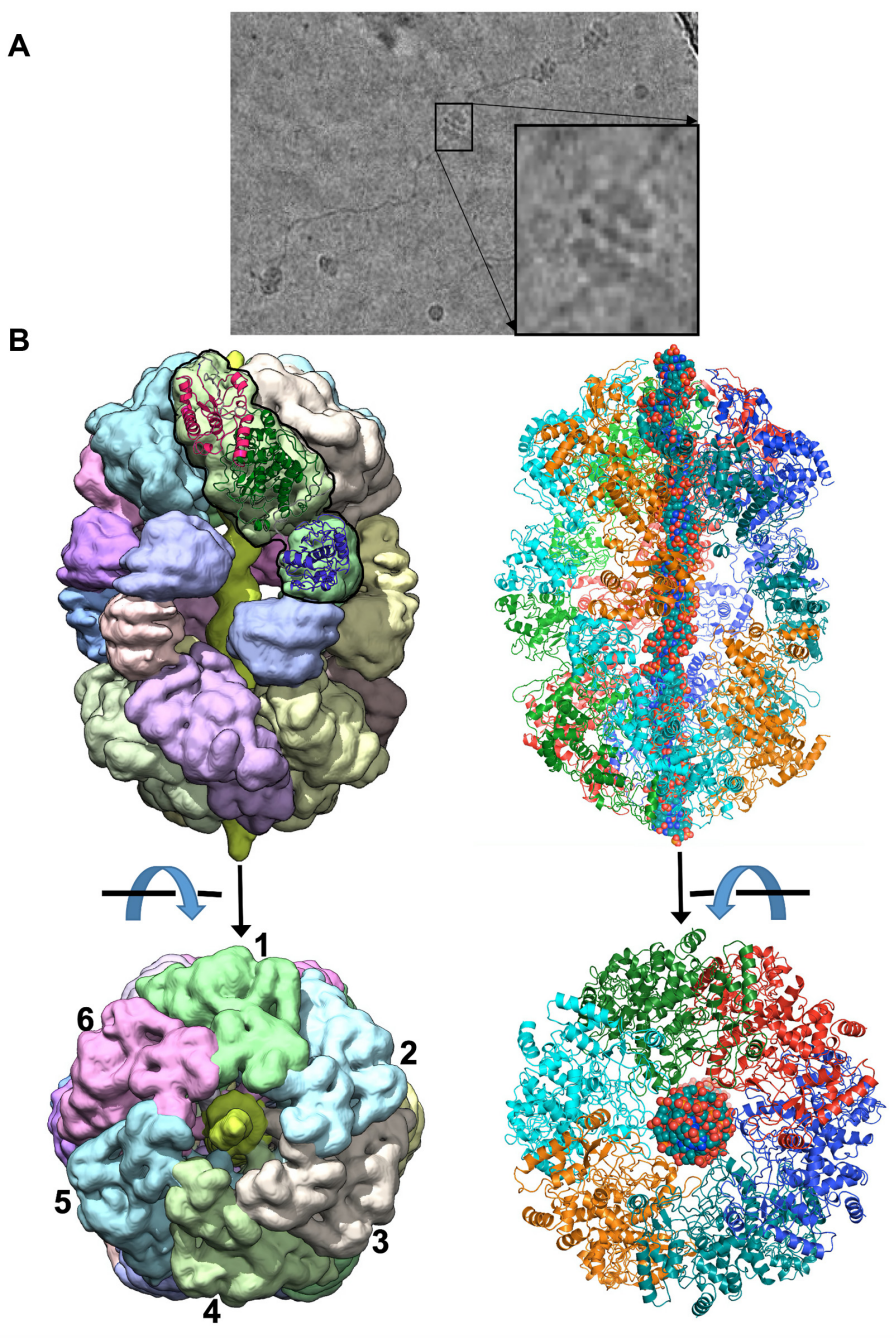
CryoEM structure of BrxL_E280Q_–DNA complex. Panel **A**: sample CryoEM micrograph of BrxL_E280Q_. See also Supplementary Figure S2b. Large symmetric particles are observed evenly distributed on the grid, with a subset of the largest particles appearing to be associated with long fibers. Panel **B**: CryoEM map corresponding to ∼3 Å resolution (*left*) and corresponding ribbon diagram of DNA-bound BrxL assemblage (*right*). The DNA-bound BrxL chamber contains 12 individual subunits, corresponding to a tail-to-tail dodecameric arrangement (a dimer of hexamers), wrapped around six full turns of DNA. DNA contacts at each end of the complex are established entirely by the NTD and central ATPase domains of each subunit. See also Supplementary Figure S4 and Supplementary Movies S1–3.

Grids generated using BrxL_E280Q_ also displayed a mixture of partially and fully assembled protein complexes, with the latter displaying protrusions from each end of the long axis of the complex. A subset of those largest particles was associated with long fibers that appeared to correspond to strands of DNA (Figure 3A). A subsequent CryoEM structure determination of full-sized particles produced electron density maps extending to ∼3 Å resolution that correspond to dodecameric particles comprising two six-fold symmetric (hexameric) protein rings, again associated with one another in an antiparallel tail-to-tail arrangement (Figure 3B and Supplementary Figure S1b).

In addition to the difference in protein copy number and symmetry between the unbound versus DNA-bound BrxL protein assemblages (corresponding to two heptamers versus two hexamers), the BrxL_E280Q_ density maps also demonstrated the unambiguous presence of double stranded DNA spanning the length of the BrxL chamber, which would seem likely to represent DNA from the bacterial expression host that was bound by BrxL during expression and/or cell lysis and then carried through the purification process. The density of the DNA was not of sufficient resolution to directly determine a specific sequence of the bound DNA moiety, and so the modeled identity of bases in the refined model represents an arbitrary sequence based on the best fit to density at each position. Approximately 6 full turns of DNA (62 basepairs) were visible and modeled into the density.

**Figure 4.**
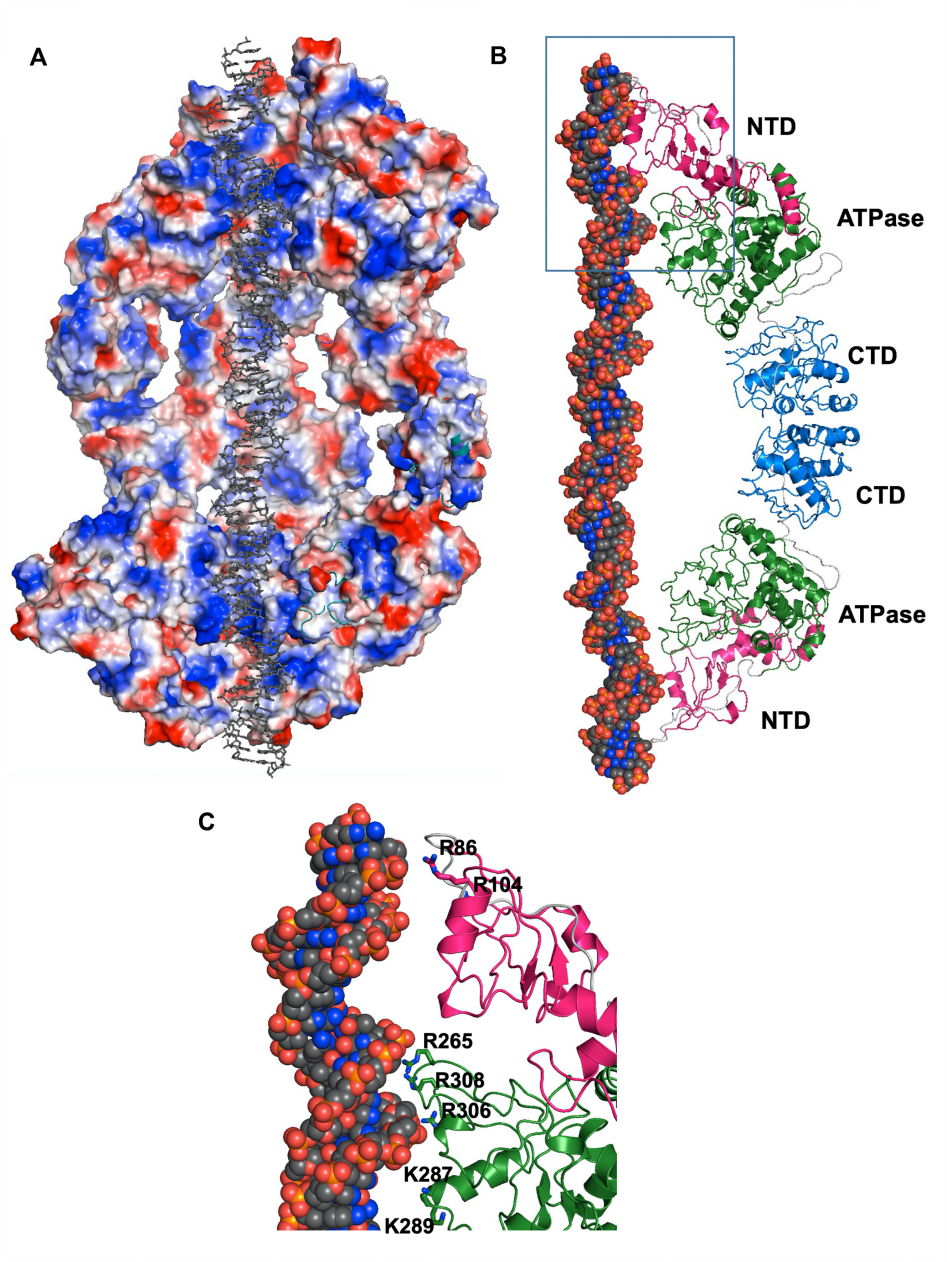
Details of the BrxL_E280Q_ assemblage and its DNA interactions. Panel **A**: electrostatic surface of the interior of the BrxL(E280Q) assemblage. One half of the entire channel is shown for clarity. The DNA is shown as grey sticks, transiting the channel. The bulk of positively charged basic residues (shown as blue) are observed near each end of the DNA (corresponding to the NTD and central ATPase domains of the protein subunits). Panels **B** and **C**: topology and DNA contact points displayed by two BrxL subunits in a tail-to-tail arrangement. The NTDs and central ATPase domains of each subunit make contact to both ends of the bound DNA. Multiple basic DNA contacting residues are localized to several loops from the two domains. The residues that appear to be involved in DNA contacts are labeled in panel C. See also Supplementary Figure S5.

The dimensions of the BrxL (E280Q) dodecamer are ∼195 Å in the longest dimension and 140 Å across the exterior diameter of the cylinder. The diameter of the interior chamber is narrowest (∼30 Å) at each end of the protein assemblage, where positively charged loop regions from the NTD and AAA+ ATPase domains of each subunit are in closest contact with the DNA (Figure 4A), and considerably wider (∼80 Å) near the chamber's equator, where the C-terminal domain of each subunit makes contacts with the same domain in the opposing protein hexameric ring. The interior of the chamber contains a substantial amount of open space such that a substantial portion of the bound DNA appears largely solvent-exposed (Figure 4B). Multiple contacts to the DNA backbone, at each end of the bound duplex, are contributed by basic residues extending from loops contributed by the NTD and the AAA+ domains (Figure 4C).

The structural rearrangements observed when comparing unbound, DNA-free heptameric BrxL_WT_ assemblages to DNA-bound BrxL_E280Q_ hexameric assemblages (Supplementary Figure S4 and Supplementary Movies S1–S3) involve (1) a hinged motion centered around a peptide linker (residues 469 to 497) connecting the AAA+ ATPase domain and the CTD, and (2) a pivot of the two CTD domains, relative to one another, in each ‘tail-to-tail’ subunit interface around the BrxL equator. The resulting conformational changes, propagated around each subunit, include movements of each N-terminal region by up to 20 Å towards the bound DNA duplex, a significant increase in the buried surface area between adjacent subunits in the DNA-bound structure (from ∼1100 Å^2^ to ∼1300 Å^2^), and an increase in the number of putative directional hydrogen bonds and salt bridges that can be modeled within each interface.

### In vitro characterization of DNA and ATP requirements for assembly of BrxL multimers

**Figure 5.**
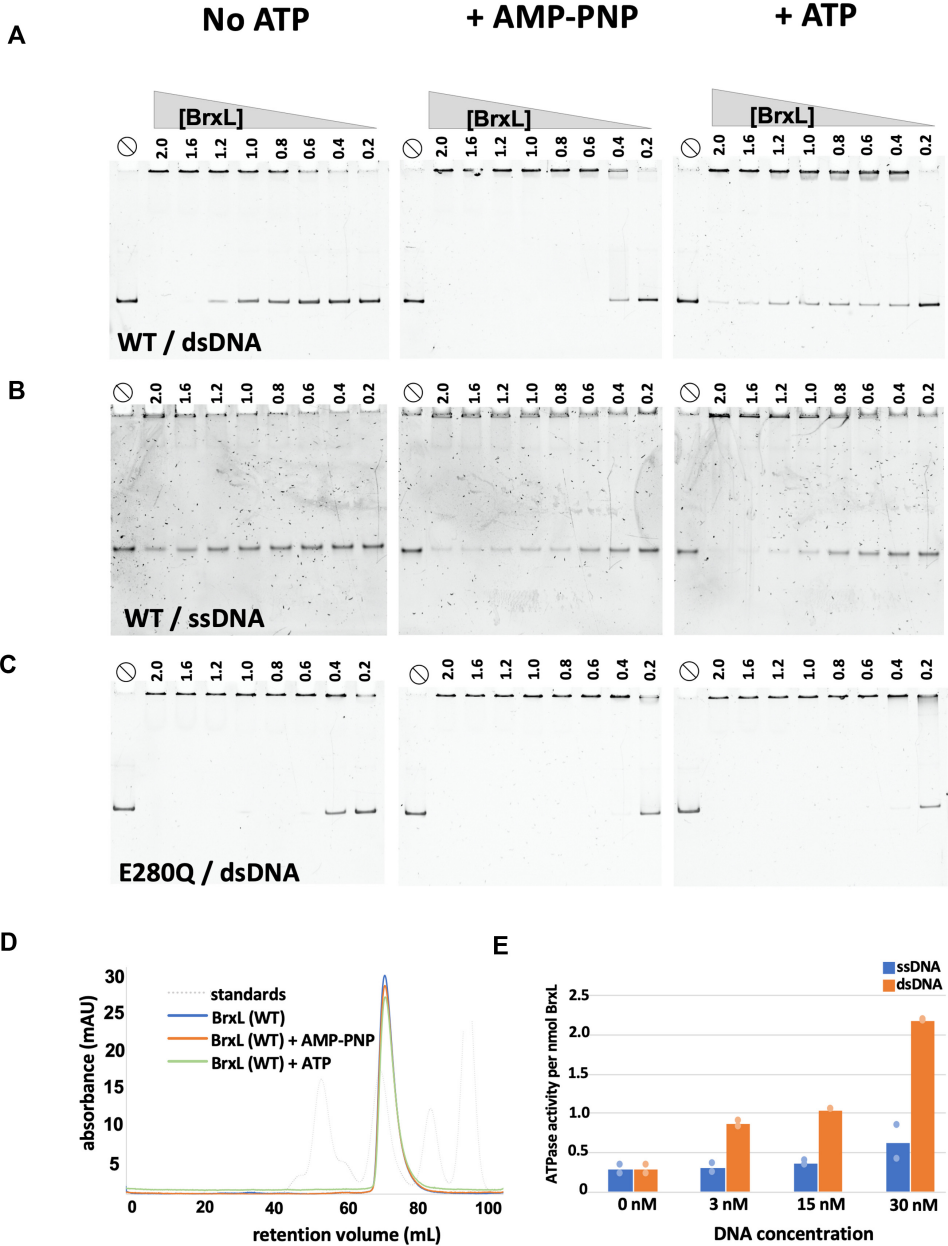
BrxL multimer assembly is ATP- and DNA-dependent. Panels **A**, **B**: EMSA analyses of BrxL_WT._ Previously isolated monomeric fractions of BrxL_WT_ from the initial SEC purification (Supplementary Figure S3a) were incubated with **(**panel A) dsDNA (207 basepairs) or (panel B) ssDNA (100 nucleotides) at the indicated concentrations (in μM). Experiments were performed with no added nucleotide (left panels), in the presence of 2.5 mM AMP-PNP (a non-hydrolyzable ATP analogue; middle panels), or in the presence of 2.5 mM ATP (right panels). Reactions were separated on native acrylamide gels and the DNA was visualized. Panel **C**: EMSA analysis of BrxL_E280Q_ incubated with dsDNA. Panel **D**: purified monomeric BrxL_WT_ was incubated without added nucleotide, with AMP-PNP (1.25 mM), or with ATP (1.25 mM) and re-run over an SEC column. All three conditions eluted with the same profile lacking early eluting peaks that would indicate renewed multimer formation. This analysis demonstrates that ATP and AMP-PNP alone are insufficient to induce multimer formation. Panel **E**: The ATPase activity of 1.5 μM BrxL_WT_ was measured in the presence of the indicated concentrations of ssDNA or dsDNA. BrxL_WT_ has low-level ATPase activity that is strongly stimulated by dsDNA (∼8-fold). These samples were run in duplicate; dots indicate individual reactions.

Having determined the structures described above, we next sought to characterize the requirements of DNA, ATP binding, and ATP hydrolysis for assembly of BrxL multimers. To address this, we examined the ability of the monomeric fraction (purified from SEC) of wild-type BrxL (BrxL_WT_) to bind to ssDNA and dsDNA probes using electrophoretic mobility shifts (EMSA or ‘gel shifts’) in the absence or presence of either ATP or its non-hydrolyzable analog AMP-PNP (Figure 5A, B). In the absence of added nucleotides, low micromolar concentrations of BrxL_WT_ produced a clean shift in the mobility of dsDNA to a significantly larger, slower migrating species that have barely entered the gel. We interpret the shifted DNA band as likely corresponding to DNA-bound multimeric BrxL complexes (which would correspond to a molecular weight of approximately 1 megadalton), although the possibility of formation of larger species or aggregates that are trapped at the edge of the gel cannot be excluded. The presence of AMP-PNP substantially enhanced multimer assembly onto DNA (producing a fully shifted complex at ∼0.6 μM protein), whereas the addition of ATP (which also caused a measurable increase in the interaction of BrxL_WT_ with DNA) failed to fully shift the probe at the highest protein concentrations used in the experiment. The increased apparent affinity in the presence of AMP-PNP indicates that binding of ATP to BrxL promotes multimer assembly on DNA. The more subtle effect of ATP (which still enhances DNA binding, but does not generate a fully shifted complex) could be caused by several possibilities: 1) ATP hydrolysis prevents complete multimer assembly; 2) ATP hydrolysis induces multimers on DNA to fall apart; or 3) multimers may assemble on dsDNA fragments and translocate until they fall off.

BrxL_WT_ has a lower apparent affinity for ssDNA than for dsDNA (Figure 5B), but still appears able to form higher order multimers. AMP-PNP did not enhance multimer formation on ssDNA to the extent that was observed with dsDNA.

We next tested multimeric assembly of BrxL_E280Q_ under the same conditions (Figure 5C). In the absence of any added nucleotide, BrxL_E280Q_ displayed a substantially higher apparent affinity for dsDNA, producing a fully shifted complex DNA at ∼0.6 μM. The binding profile mirrors that of BrxL_WT_ in the presence of AMP-PNP. The addition of AMP-PNP and ATP further increased the BrxL_E280_ apparent binding affinity, completely shifting dsDNA at 0.4 μM protein. These results are consistent with BrxL_E280Q_ purifying in a DNA-bound form. We believe that since BrxL_E280Q_ is unable to hydrolyze ATP, it behaves in a similar manner to BrxL_WT_ incubated with a non-hydrolyzable ATP analog.

We further examined the effect of ATP on BrxL_WT_ and BrxL_E280Q_ on multimer formation via SEC (Figure 5D and Supplementary Figure S5). In these analyses, each purified monomeric BrxL construct was independently incubated with or without ATP or AMP-PNP and DNA and re-run on SEC; peaks eluting with earlier retention times indicate the formation of multimeric complexes. Consistent with our EMSA analyses, BrxL_E280Q_ incubated with DNA, as well as BrxL (WT) incubated with ATP and DNA, produced early eluting peaks (Figure S5). However, BrxL_WT_ incubated with ATP or AMP-PNP (but no DNA) eluted entirely as monomers and lacked early eluting peaks (Figure 5D), demonstrating that either nucleotide by itself is not sufficient to induce BrxL multimer formation. Considered together, these results suggest that BrxL multimer assembly requires both binding of ATP to BrxL and the presence of DNA.

### dsDNA stimulates BrxL ATPase activity

We next measured BrxL_WT_ ATPase activity in the absence and presence of ssDNA and dsDNA as described in Methods. Activity was measurable and found to be stimulated by dsDNA by ∼8-fold (Figure 5E). In contrast, ssDNA induced a more modest, approximately 2-fold increase in activity.

### Analysis of BrxL mutants

**Figure 6.**
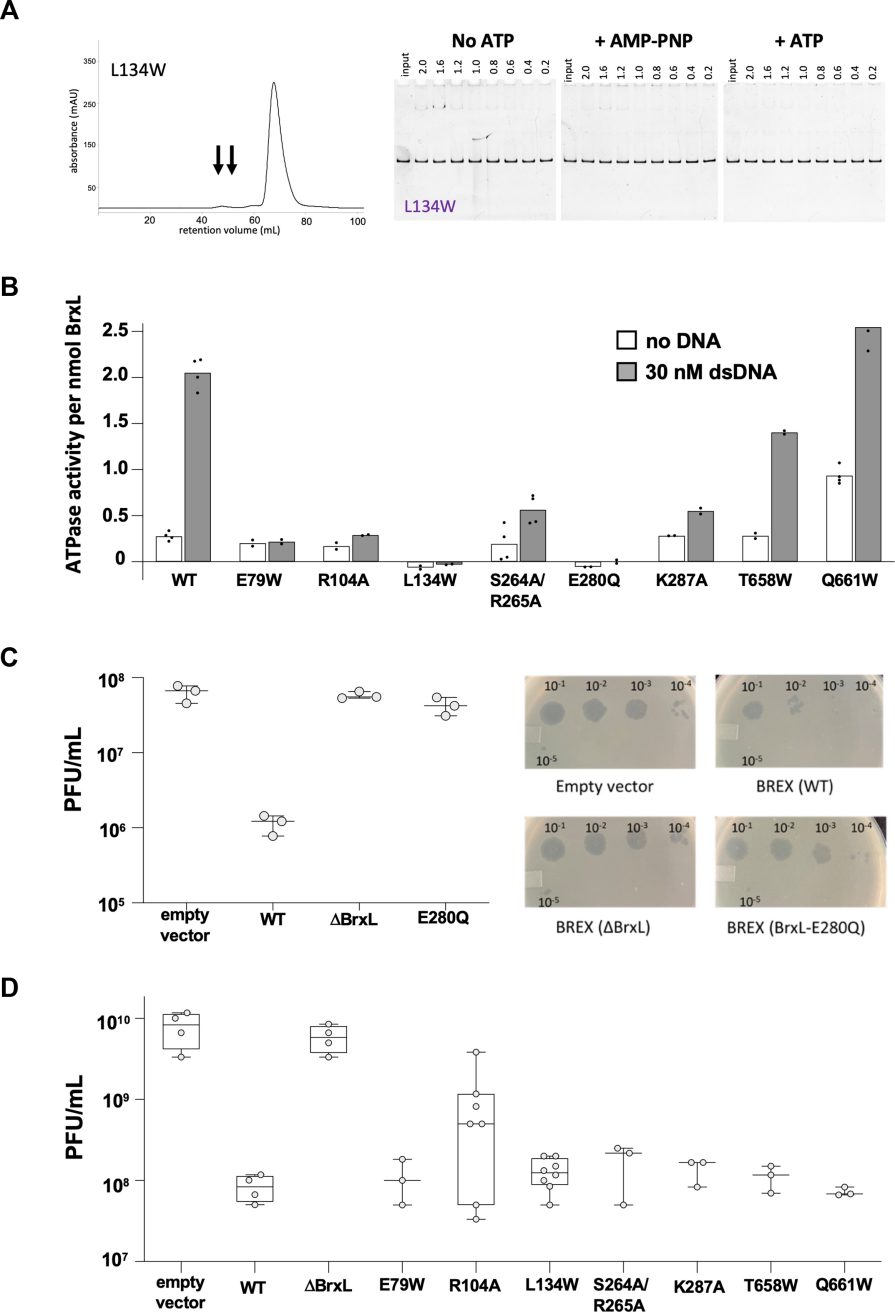
Analysis of BrxL mutants. Panel **A**: Initial SEC elution profile (left panel) and EMSA analysis (right panels, under the same conditions described for BrxL_WT_ in Figure 5) of BrxL_L 134W_. Both analyses were carried out for an additional seven BrxL mutants as shown in Supplementary Figures S7 and S8). These results show that the L134W mutant is deficient in multimer assembly and binding on DNA. Panel **B**: The ATPase activity of the indicated BrxL constructs were determined in the absence and presence of dsDNA (30 nM). Dots represent measurements of individual reactions; height of bars represents the mean values of those individual reactions. Panel **C**: Relative phage restriction activities, as a function of the presence, absence, or mutation of the BrxL gene in the BREX operon, were evaluated by determining the fold change in phage plaque formation using λ_vir_ phage and *E. coli* strain ER2683. For each construct tested, we performed a minimum of three (and up to eight) biological replicate experiments, each of which corresponded to two separate technical replicates. Each data point corresponds to the average of technical replicates within a single biological replicate. Horizontal bars are the median of the biological replicates; vertical bars indicate the maximum and minimum readings. Whereas the intact BREX operon (including wild-type BrxL) reduced phage plaque formation efficiency by approximately two orders of magnitude, a precise deletion of the BrxL gene (‘ΔBrxL’) or the introduction of the E280Q mutation into the BrxL ATPase Walker B motif (‘BrxL_E280Q_’) resulted in phage plaque formation activity comparable to when the BREX system is entirely absent (‘Empty Vector’). The right panel shows representative plaque formation plates used for generating data. Panel **D**: Relative phage restriction efficiencies for additional BrxL mutants. Plots are displayed as described for panel C, with the additional inclusion of boxes representing 25th and 75th percentile of standing in the group for individual measurements. Data plots generated using PRISM with box and whisker plots generated according to standard program method.

Using the DNA-bound structure as a guide, we generated eight additional BrxL constructs, corresponding to seven individual point mutations and one double mutant, at amino acid residues likely to be important to different aspects of BrxL functionality. These included (i) four mutations in the pore of the hexameric chamber in proximity to DNA (R104A; S264A/S265A; K287A); (ii) two mutations at positions that mediate contacts between protein subunits in each hexameric ring (E79W, L134W); (iii) two residues in the C-terminal domain that mediate dimerization between hexamers (T658W,Q661W) and (iv) the original E280Q mutation in the ATPase active site (Figure 6 and Supplementary Figure S6).

We purified each mutant construct over metal affinity chromatography and SEC as previously described and compared their solution behavior during the second chromatographic step to BrxL_WT_ protein. E79W, L134W, R104A and S264A/R265A show significantly reduced peaks at early retention volumes, consistent with disruption of multimeric complexes (Figure 6A and Supplementary Figure S7). T658W displayed a substantial shift toward multimers and has a monomeric peak that elutes at a slightly later retention volume than other BrxL constructs. Q661W also displays an increased fraction of multimers.

Having visualized the effect of these mutations on solution behavior via SEC, we went on to examine each construct's ability to (1) assemble as a bound complex on dsDNA; (2) display DNA-dependent ATPase activity, and (3) support phage restriction in the context of the intact BREX operon.

*The comparative EMSA assays* (Figure 6A and Supplementary Figure S8) demonstrated that several of the mutated constructs, located either in the N-terminal protein-protein interface (L134W) or in the DNA-contacting pore (R104A, S264A/R265A and K287A) substantially reduced dsDNA binding. In contrast, two mutations in the dimer interface (T658W and Q661W) bound DNA in a similar manner to wild-type BrxL, although the addition of ATP did not appear to influence the production of fully shifted complexes as observed with wild-type BrxL.

*The comparative ATPase activity assays* of each mutant in the presence and absence of dsDNA (Figure 6B) demonstrated that the L134W and E280Q mutants had no detectable activity in either condition. Conversely, mutations in the dimer interface (T658W or Q661W) retained or even increased ATPase activity, with the basal activity of Q661W appearing to be elevated relative to WT. The remaining mutant constructs displayed decreased ATPase activity, although not to the extent of the E280Q mutation located in the ATPase Walker B catalytic motif.

*The comparative phage restriction assays* were performed using a plaque formation assay (further described in Materials and Methods and Supplementary Methods) using *E. coli* strain ER2683 transformed with a pACYC plasmid harboring a full BREX system. The strain was challenged with serial 10-fold dilutions of a virulent mutant of the phage λ (λ_vir_) that is unable to undergo lysogeny. In this assay (Figure 6C, D), BREX containing WT BrxL reduced plaque formation by approximately two orders of magnitude relative to *E. coli* transformed with an empty plasmid. Disruption of the ATPase active site via the E280Q mutation significantly reduced BREX activity to the same extent as precise deletion of BrxL altogether. Of the remaining mutants, only R104A demonstrated a reduced ability to restrict phage (∼10-fold), whereas the remaining six mutants behaved similarly to wild-type BrxL.

## DISCUSSION

In this study we have demonstrated that the BrxL phage restriction factor is a multimeric DNA binding protein that displays ATP-dependent assembly on double stranded DNA. BrxL’s N-terminal and AAA + ATPase domains mediate heptamerization (in the absence of bound DNA) or hexamerization (in the presence of bound DNA). The C-terminal domain mediates further dimerization of either assemblage into a tail-to-tail dimer of protein rings.

BrxL displays considerable diversity in its solution behavior and assembly properties, corresponding to an apparent equilibrium between various assemblage states. Lon protease (LonP), which selectively degrades mutant and abnormal proteins in bacteria and in eukaryotic mitochondria, behaves similarly, having also been observed in both hexameric (34,35) and heptameric (36) assemblages, and to participate in a dynamic equilibrium with monomers and smaller oligomeric subcomplexes. Additional studies have suggested that ATP binding is associated with changes in Lon protease conformations that influence the fraction of protein found in larger symmetric assemblages (30). Similarly, archaeal RadA and its bacterial and eukaryotic homologues RadA and Rad51, which facilitate homologous recombination during double strand break repair, have also been visualized in both hexameric (20,37,38) and heptameric (39) assembly states in the presence and absence of double stranded DNA, as well as in helical filaments wound around single stranded DNA (40). At least one of those studies have suggested that the difference between hexameric and heptameric assemblages may correspond to activated DNA-bound and inactive DNA-free protein states (39).

Whereas all of the mutations introduced into the BrxL NTD and ATPase domains (either in the protein subunit interface or in the DNA-contacting pore region, or the ATPase active site) are associated with altered behavior in at least one *in vitro* assay (assembly on DNA and/or ATPase activity), only the catalytically inactivating E280Q mutation (in the ATPase Walker B motif) clearly reduced phage restriction activity. That result implies that other than the effect of inactivating ATPase activity, other single mutations in our studies can be accommodated and rescued in a living cell armed with an otherwise intact BREX system.

Perhaps the most surprising of the mutants to still promote phage restriction is BxrL (L134W), which did not form multimeric complexes with dsDNA (in EMSA assays) and displayed no detectable ATPase activity, even in the presence of dsDNA. We envision two possibilities that could explain this result. First, it is possible that our *in vitro* assays do not accurately reflect the process by which BrxL multimer assembly occurs in cells. Replicative DNA helicases typically require other loading factors that direct an orchestrated set of steps that result in loading of helicases onto DNA. It is possible that other factors, perhaps including BREX components, perform a similar function in loading BrxL onto DNA *in vivo*. A second possibility is that monomeric BrxL, rather than multimeric BrxL assemblages, is the form of BrxL of that participates in restriction. In this context, perhaps monomeric BrxL interacts with other BREX components to activate their phage restriction capabilities.

The C-terminal domain of BrxL displays distinct solution behavior from the structurally similar LonP_C_ and RadA_C_ domains in the context of assembled BrxL complexes. Isolated LonP_C_ and RadA_C_ domains form hexamers in solution and can bind their corresponding substrates in the central pore (20,41). In contrast, purified BrxL_C_ is monomeric in solution and (in the context of full-length BrxL) appears to mediate dimerization of heptameric (without ATP) or hexameric (with ATP) complexes through protein-protein interactions with another BrxL_C_ partner. In the DNA-bound dodecameric complex, BrxL_c_ domains are far removed from dsDNA occupying the central pore and do not share significant conservation with LonP residues essential for protease activity (19,29,30,41) or with RadA residues involved in DNA binding (20,38) (Supplementary Figure S1b). These observations imply that BrxL’s CTD has evolved a distinct functional role from the LonP_C_ and RadA_C_ domains.

The behavior of the Q661W mutation may indicate an additional, perhaps regulatory, role for BrxL’s CTD. BrxL_Q661W_ displayed substantially higher basal ATPase activity compared to BrxL_WT_ (∼3.3-fold), which was stimulated to a greater extent than BrxL_WT_ in the presence of dsDNA. In EMSA assays, BrxL_Q661W_ had a lower apparent affinity for dsDNA (in the presence of ATP) compared to BrxL_WT_, which may be a consequence of its higher ATPase activity. These results are consistent with the CTD regulating BrxL ATPase activity until it properly assembles on DNA.

The mechanism by which type 1 BREX systems restrict phage is not yet understood but occurs at a step prior to phage replication and does not involve cleavage of the phage genome (12). BREX systems presumably employ a DNA scanning mechanism that differentiates methylated (host) from unmethylated (foreign) sites; consistent with this, the *Salmonella enterica* BREX system (StySA) is observed to much more effectively restrict phage containing a higher number of unmodified PglX recognition sequences (21). Furthermore, given that all six or seven conserved genes in various type I BREX systems so far characterized, including BrxL, are required for restriction, it seems likely that a multi-protein BREX complex carries out this scanning function. In the StySA BREX system, the authors identified a BrxC mutant containing a handful of relatively conserved mutations in its C-terminal domain that is competent for methylation but deficient for restriction (21). They proposed that BrxC’s C-terminal domain is critical for scanning and identifying unmethylated target sites and perhaps for licensing BrxL for phage restriction.

AAA+ ATPases comprise an ancient protein family that has evolved into distinct clades that are functionally associated with a wide range of cellular activities, including modification of proteins and nucleic acids (42). To better understand BrxL’s relationship with the AAA+ ATPase family, we used the DALI server (28) to search for structural homologs of BrxL’s AAA+ ATPase region (residues 157–469). This analysis identified numerous MCM proteins, which comprise the replicative helicase in archaea and eukaryotes (43), as having the closest structural homology to BrxL across its AAA+ ATPase domains. We further observed that a region in BrxL’s NTD (amino acids 71–157) shared structural homology to MCM proteins outside of the AAA+ ATPase domains. Over ∼330 amino acids, BrxL’s NTD+ ATPase shares an rmsd with the archaeal MCM from *Saccharolobus solfataricus* of ∼4 Å across backbone atoms (Supplementary Figure S9). MCM proteins act as replicative helicases in archaea and eukarya and also assemble into dimers of hexamers on origins of replication, albeit in an opposite subunit organization relative to BrxL; in MCM assemblages their N-terminal domains face each other and promote dimerization (43–45), whereas in BrxL their C-terminal domains play that structural role. During the course of this study, we systematically examined the ability of BrxL to act as a DNA helicase or strand exchange factor but did not succeed in conclusively demonstrating either activity. This question is still an open area for further experimentation.

Motivated by the observation that BrxL harbors structural similarity to MCM proteins, we examined whether additional type 1 BREX factors contain motifs associated with DNA replication factors by generating structural predictions using the AlphaFold server (46) and submitting those predictions to DALI. This analysis indicated that BrxC contains an AAA+ ATPase domain that shares closest structural homology to origin recognition complex (‘ORC’) subunits across this domain (Supplementary Figure S10). ORC factors, along with the eukaryotic gene CDC6 and bacterial protein DnaA, comprise a clade of AAA+ ATPases associated with the initiation of DNA replication (42). In eukaryotes and archaea, the 6-subunit ORC complex binds to origins of replication in G1 phase, and along with Cdc6 and Cdt1 sequentially loads a pair of MCM hexamers onto origins of replication (43,47,48).

The same analysis further indicated that BrxB contains a AAA+-like fold with structural homology to ORC and DnaA proteins (Supplementary Figure S11); across ∼130 amino acids, BrxB shares ∼4.0 Å rmsd across backbone atoms within the AAA+ ATPase region of DnaA. This finding was surprising, as BrxB was not previously reported to contain a AAA+ fold. BrxB lacks conserved residues in the Walker A and Walker B regions, indicating it likely is deficient in ATP binding if it does indeed represent a new member of the AAA+ protein family. Among eukaryotic ORC proteins, Orc2 and Orc3 family members also have AAA+-like domains that lack conserved features of this domain. We further identified that the BREX type 3 factor annotated as BrxF (9) also contains a AAA+ motif belonging to the ORC/DnaA clade and may be a functional homolog of BrxB. Models of BrxB and BrxF proteins superpose well (rmsd of 3.6 over 146 residues), and type 3 systems lack an annotated BrxB factor. Finally, the BrxD factor present in type 2 and type 6 BREX systems also contains a AAA+ ATPase domain of the ORC clade.

It is therefore tempting to speculate that multiple factors within various BREX systems use similar structural motifs to jointly recognize and then interact or interfere with origins of replication in phage, via a mechanism that requires the presence of unmodified PglX target sites to license their activity, eventually loading BrxL onto DNA as part of a mechanism that ultimately inhibits phage DNA replication. Many phage utilize DNA replication mechanisms that involve initial assembly of a replisome, usually with local DNA unwinding, at a defined replication origin (49). DNA replication for the phage used in this study (λ_vir_) begins approximately 10 minutes post infection at a unique origin within the phage genome, via a mechanism requiring a specific *ori* site on the phage chromosome, which is recognized in *trans* by the phage-coded O and P proteins (50); it is equally tempting to speculate that process being a target for disruption or interference by the BREX system. Future studies are needed to determine requirements for more robust activity, as well as how this activity may contribute to phage restriction.

## DATA AVAILABILITY

The sequences of BrxL and its associated protein factors from the *Acinetobacter* were previously described in (14). The sequence of the entire BREX operon is deposited at GenBank with protein ID WP_176538600.1. The *Acinetobacter* assembly accession ID is ASM1337479v1, leading to all 15 genome sequences (chromosome (CP055277.1) and corresponding plasmids. The *Acinetobacter* genome sequencing BioProject number is PRJNA638470 (BioSample number SAMN15195663; txid number txid2743575). The crystal structure of BrxL_C_ has been deposited in the RCSB database (PDB ID code 8EIL). The CryoEM models and corresponding maps for free and DNA-bound BrxL have been deposited in the RCSB database (ID codes 8EMC and 8EMH respectively) and in the EMDB (ID codes EMD-28244 and 28248, respectively). The original source data and raw images corresponding to the biochemical analyses of BrxL function and activity have been uploaded to the Harvard Dataverse public repository (https://dataverse.harvard.edu/dataverse/BrxL).

## Supplementary Material

gkad083_Supplemental_Files
