# Supplementary material for: Structure, substrate binding and activity of a unique AAA+ protein: the BrxL phage restriction factor
Source: Nucleic Acids Res. 2023 Feb 16;51(8):3513–28. doi: 10.1093/nar/gkad083 (PMC10164562; doi:10.1093/nar/gkad083)

Supplementary Materials

Structure, substrate binding and activity of a unique AAA+ protein: the BrxL phage restriction factor

Supplementary Methods

Identification and subcloning of a novel type I BREX system **(Duplicated from (1)).** "Whole genome sequencing of *Acinetobacter* sp. NEB394 was carried out using Pacific Biosciences (PacBio) SMRT sequencing technology (2). The assembled genome and associated plasmids have been submitted to NCBI (NCBI:txid2743575). Putative DNA modification motifs were identified using PacBio SMRTAnalysis 2.3.0 Modification_and_Motif_Analysis_1.0. To assign methylation function to DNA methyltransferase genes, putative DNA methyltransferase genes identified within REBASE (3) and flanking genes were examined using the standard protein blast (BLASTP) program from NCBI (4). One putative N6A DNA methyltransferase appeared as part of a classic BREX system with flanking genes closely related to the BrxA, BrxB, BrxC, PglZ, and BrxL genes (5).

Oligonucleotide primers used in these studies (see tables at end of **Supplementary Methods** were synthesized by Integrated DNA Technology (Coralville, IA). Molecular biology reagents including Q5 Hot Start High-Fidelity DNA polymerase, NEBuilder HiFi DNA assembly master mix, restriction enzymes, DNA size standards, and competent cells were provided by New England Biolabs. Genomic DNA isolation, plasmid purification, agarose gel and PCR clean ups were performed using Monarch DNA kits (NEB). Plasmid DNA constructs were confirmed by sequencing on the ABI 3130xl capillary machine (Applied Biosystems) and PacBio RSII (Pacific Biosciences).

The 14.1kb BREX operon was PCR amplified as three ~4.7 kB fragments from genomic DNA using overlapping primers designed for NEBuilder HiFi assembly. The expression plasmid pACYC184 (6) was amplified by inverse PCR to insert the BREX operon downstream of the Tet promoter. The *Acinetobacter* sp. NEB394 BREX system was subcloned in the pACYC184 expression vector under the control of a constitutive Tet promoter, using NEBuilder HiFi DNA assembly mix. Briefly, PCR-amplified insert and plasmid DNAs were mixed in equimolar ratios, incubated at 50°C for 60 minutes, and transformed into ER2683 competent cells per manufacturer's instructions. Individual colonies were selected and grown overnight in LB broth (10 g/L soy peptone, 5 g/L yeast extract, 5 g/L NaCl, 1 g/L $MgCl_2$, 1 g/L dextrose) supplemented with chloramphenicol (25 μg/ml). Total DNA was isolated from overnight cultures and prepared for sequencing on PacBio RSII (Pacific BioSciences, Menlo Park, CA).

Individual colonies were grown overnight in LB broth supplemented with chloramphenicol (25 μg/ml). Plasmid DNAs were isolated from overnight cultures for analysis by restriction digestion and PacBio Sequel II sequencing of the entire plasmid. The size of plasmid constructs was determined by digestion with HpaI in a 30 µl reaction containing 5 ng template DNA, 1x NEB CutSmart Buffer, and 1 µl restriction enzyme, at 37°C for 30 minutes. Digestion products were analyzed by agarose gel electrophoresis. Insertion of the 93 basepair sequence was verified via Sanger sequencing (ABI 3130xl capillary

machine) and the entire construct sequence (~17 kb) was confirmed via sequencing on the PacBio Sequel II system (Pacific Biosciences)."

BrxL Gene Deletion and point mutations **(Duplicated from (1)). "**The BrxL gene was precisely removed from the cloned BREX operon (pAcBREX_WT) by inverse PCR. The pAcBREX_WT was amplified using Q5 Hot Start High-Fidelity DNA polymerase (NEB) and primers containing a 15-21 base overlap targeting DNA sequences immediately adjacent to the 5' and 3' ends of the gene to be removed. Following amplification, template DNA was removed by digestion with DpnI for 30 minutes at 37°C. PCR products were analyzed by agarose gel electrophoresis and purified using Monarch gel extraction or PCR purification kits. Purified amplicons were self-ligated using NEBuilder HiFi DNA Assembly Master Mix and transformed into ER2683 competent cells to generate a plasmid construct lacking the BrxL gene (pAcBREX_ΔBrxL).

Individual colonies were grown overnight in LB broth supplemented with chloramphenicol (25 μg/ml). Plasmid and total DNAs were isolated from overnight cultures for analysis by restriction digestion and PacBioRSII sequencing respectively in a 30 μl reaction containing 0.5 ng template DNA, 1x NEB CutSmart Buffer, and 1 μl restriction enzyme, at 37°C for 30 minutes. Digestion products were analyzed by agarose gel electrophoresis. The ligation junction for plasmids producing the predicted restriction patterns were confirmed via Sanger sequencing (ABI 3130xl capillary machine). Upon confirmation by restriction digestion and Sanger sequencing, total DNA derived from the corresponding overnight culture was used to generate PacBio libraries analyzed for sequence confirmation of the expression plasmid."

Bacterial growth and phage restriction assays **(Duplicated from (1))***.* "All infections using phage λ were performed with a virulent mutant of the phage unable to undergo lysogeny (λ_{vir}) (7). In all experiments, cells were transformed with pACYC-based plasmids encoding the wild-type BREX operon, various deletion mutants, or control vectors that lack the BREX operon. Transformants were stored at -80°C as glycerol stocks. Experiments were performed both in *E. coli* NEB 5-alpha cells (New England Biolabs) and in *E. coli* strain ER2683 (New England Biolabs).

All experiments began using isolated colonies derived from freezer stocks, which were then used to inoculate 4 ml of lysogeny broth (LB; 10 g/L casein peptone, 10 g/L NaCl, 5 g/L ultra-filtered yeast powder) supplemented with 1.25 mM $MgCl_2$, 1.25 mM $CaCl_2$, and 25 mg/ml chloramphenicol ('overnight culture media'). Cultures were grown overnight at 37°C with shaking at 220 rpm. The next day, overnight cultures were diluted 50-fold in 4 ml of LB supplemented with 1.25 mM $MgCl_2$, 1.25 mM $CaCl_2$, 0.2% maltose, and 25 μg/ml chloramphenicol ('outgrowth media') and grown to mid-log (absorbance readings at 600 nm varied between 0.2 and 0.6) at 37°C, shaking at 220 rpm. Mid-log cultures were then diluted to an OD_{600} of 0.01 in LB containing 1.25 mM $MgCl_2$, 1.25 mM $CaCl_2$, 25 mg/ml chloramphenicol. Phage titers were determined via a plaque assay to ensure accurate MOIs. Phage λ_{vir} samples were serially

diluted in SM buffer (50 mM Tris-HCl, 25 mM NaCl, 4 mM $MgSO_4$) before use and added to the bacterial samples at multiplicities of infection (MOIs) ranging from 0.001 to 1.0. SM buffer alone was added to samples without phage. Cultures were then arrayed in triplicate across a 96-well plate (Greiner cat#655083, 100 µl per well) and grown for ten hours in a BioTek Cytation three plate reader at 37°C with continuous orbital shaking at 282 cycles per minute. Absorbance readings were taken every 15 minutes at 600 nm.

Additional, complementary phage plaque formation assays were performed using the same titred phage stock and overnight cultures of ER2683 grown in overnight culture media. Multiple dilutions of the overnight cultures were generated the next morning (between 30- and 100-fold) in outgrowth media and then grown to an OD_{600} of approximately 0.4 to 0.5. 80 microliters of each sample were mixed with 3 mL of top agar (0.5% agar in LB supplemented with 1.25 mM $MgCl_2$, 1.25 mM $CaCl_2$, and 25 µg/ml chloramphenicol) and applied to bottom agar plates (1.5% agar in LB supplemented with 1.25 mM $MgCl_2$, 1.25 mM $CaCl_2$, and 25 µg/ml chloramphenicol). Plates were allowed to solidify and dry for approximately 15 minutes and then spotted with 5 µl of 10-fold serial dilutions (ranging from 10^1 to 10^8 dilutions) of titred phage. Plates were incubated overnight at 37°C and examined the following morning for plaque formation. Each experiment was performed with a minimum of three biological replicates."

REFERENCES

1. Luyten, Y., Hausman, D., Young, J., Doyle, L.A., Ubilla-Rodriquez, N.C., Lambert, A.R., Arroyo, C., Forsberg, K., Morgan, R.M., Stoddard, B.L. *et al.* (2021) Identification and characterization of BrxR as a regulatory gene in the BREX phage restriction system. *bioRxiv*, 2021.2012.2019.473356.
2. Eid, J., Fehr, A., Gray, J., Luong, K., Lyle, J., Otto, G., Peluso, P., Rank, D., Baybayan, P., Bettman, B. *et al.* (2009) Real-time DNA sequencing from single polymerase molecules. *Science*, **323**, 133-138.
3. Roberts, R.J., Vincze, T., Posfai, J. and Macelis, D. (2015) REBASE--a database for DNA restriction and modification: enzymes, genes and genomes. *Nucleic Acids Res*, **43**, D298-299.
4. Altschul, S.F., Gish, W., Miller, W., Myers, E.W. and Lipman, D.J. (1990) Basic local alignment search tool. *J Mol Biol*, **215**, 403-410.
5. Goldfarb, T., Sberro, H., Weinstock, E., Cohen, O., Doron, S., Charpak-Amikam, Y., Afik, S., Ofir, G. and Sorek, R. (2015) BREX is a novel phage resistance system widespread in microbial genomes. *EMBO J*, **34**, 169-183.
6. Chang, A.C. and Cohen, S.N. (1978) Construction and characterization of amplifiable multicopy DNA cloning vehicles derived from the P15A cryptic miniplasmid. *J Bacteriol*, **134**, 1141-1156.
7. Bailone, A. and Galibert, F. (1980) Nucleotide sequence of the operators of lambda ultravirulent mutants. *Nucleic Acids Res*, **8**, 2147-2164.
8. Meagher, M., Epling, L.B. and Enemark, E.J. (2019) DNA translocation mechanism of the MCM complex and implications for replication initiation. *Nat Commun*, **10**, 3117.

SUPPLEMENTARY TABLE S1. DNA oligonucleotide sequences used in study

**Subcloning into pACYC**		
DB5	GTAGGCTACGCTCTATCGTTAATTCGT	Along with DB9, amplifies pACYC backbone.
DB6	GGATCCCCGGGGAAGATCTA	Along with DB10, amplifies pACYC backbone.
DB7	AACGATAGAGCGTAGCCTACATGGAATCTG CTAACGATAAAGA	Along with DB8, amplifies BrxL coding sequence.
DB8	TAGATCTTCCCCGGGGATCCTTAATCCACC CCTAACCC	Along with DB7, amplifies BrxL coding sequence.
DB9	GTTTTGTCAAACGCCCTTATGGCTG	Along with DB5, amplifies pACYC backbone
DB10	CAGCCATAAGGGCGTTTGACAAAAC	Along with DB6, amplifies pACYC backbone
**EMSA Assays**		
gBlock_BrxR_ upstream	GCGGAGTCCATCCAAACATCGGGAGATTTT GATCAGATATGTTTAACCTGCCCTCACAAT ACCGTAAAAATAATTTACTGTATATTCATTG TAGGGTAGTCTTGATCACATTGTTTATTGAC CGATTGTTCTATGACAGCAGACAAGCATGA AGTGCTTCTCAGAATGAGGGCGATTGAGTT ACTGGCCTACTGGGAAGGGCGTTTG	template used to generate 207 bp dsDNA used in EMSA and ATPase assays.
BrxR_-132_fwd	GCGGAGTCCATCCAAACAT	PCR primer to amplify 207 bp DNA.
BrxR_+75_rev	CAAACGCCCTTCCCAGTAG	PCR primer to amplify 207 bp DNA.
Ovio2	ACTTGATTCTGTCGCTACTGATTACGGTGC TGCTATCGATGGTTTAACGTCATAGACGAT TACATTGCTACATGGAGCTGTCTAGAGGAT CCGACCCTAG	ssDNA used in EMSA and ATPase assays.

Supplementary Movie Legends

Movie S1. Conformational change of two BrxL subunits, interacting in a tail-to-tail arrangement and spanning the length of the dodecameric assemblage, from the unbound heptameric assemblage to the DNA-bound hexameric assemblage. The DNA found the latter structure is shown as a grey surface rendering for context. Image produced using the Morph function in PYMOL.

Movie S2. Conformational change of 12 BrxL subunits from the unbound heptameric assemblage to the DNA-bound hexameric assemblage. Viewed from the side of the protein chamber. The DNA in the latter structure is shown as a grey surface rendering for context. The gap between subunits at the beginning of the movie corresponds to the position of two addtional subunits in the DNA-free heptameric assemblage. Image produced using the Morph function in PYMOL.

Movie S3. Conformational change of 12 BrxL subunits from the unbound heptameric assemblage to the DNA-bound hexameric assemblage. Viewed from the end of the protein chamber. The DNA in the latter structure is shown as a grey surface rendering for context. The gap between subunits at the beginning of the movie corresponds to the position of two addtional subunits in the DNA-free heptameric assemblage. Image produced using the Morph function in PYMOL.

Supplementary Figure Captions

Figure S1. Flow chart describing cryoEM analysis for $BrxL_{WT}$ in the absence of bound DNA (S1a) and DNA-bound $BrxL_{E280Q}$ (S1b).

S1a (Unbound wild-type). ***Panels a & b:*** Two datasets at slightly different pixel size (1.16 and 1.12 Å/pix) were collected and processed separately. The selected particles from each dataset were re-extracted using extraction box sizes suggested by the script boxscaler.py. The higher resolution data set was extracted with a box size 406 pixels, Fourier cropped to 392 pixels and combined with particles selected from dataset a. ***Panel c:*** Template images of selected particles from 2D classification of the combined particles from datasets a. and b. ***Panel d:*** Results from a four-model *ab initio* 3D reconstruction using particles from c. All classes contained partial models, except Class_003 which clearly showed a completed cage of approximate 7-fold symmetry. ***Panel e:*** Result from Homogenous, Non-uniform, and Local refinements of Class_003 showing three different orientations of an hallow cage with 7-fold symmetry and corresponding Gold Standard Fourier Shell Correlation (GSFSC) of Local refinement. Similar refinements with D7 symmetry did not improve the resolution of the GSFSC. ***Panel f:*** Local resolution of the refined particle in three orientations.

S1b (DNA-bound E280Q). ***Panel a*****:** Pre-processing of a dataset collected at the Pacific North CryoEM Center (PNCC) (see table 1 for microscope and data collection parameters). ***Panel b***: Images of all classes after two rounds of 2D classification & Select 2D. ***Panel c:*** Two different orientations of models from a 4-model *ab-initio* 3D reconstruction using particles from b. Classes_0, 1 and 3 showing partial particles at various stages and closed to 50% of particles belonged to Class_002 which showed a filled cage with approximate 6-fold symmetry. However, inspection of the model clearly showed the presence of double-strand DNA in the cage. Hence further refinements of class_002 were conducted with C1 symmetry. ***Panel d***: Heterogenous refinements of class_002 from *ab-initio* 3D reconstruction. Class_000 of results from heterogenous four-model refinement of the ab initio model class_002 constituted 67% of the particles from Class_002 of *ab-initio* reconstruction and was used for final refinement. ***Panel e*****:** Two different orientations of final density map after Homogenous, non-uniform, and Local refinements; ***Panel f*****:** Two different orientation of ribbon model of dodecamer and double strand DNA with random sequence. ***Panels g & h*****:** GSFSC and Viewing direction distribution from Local refinement of map in e. ***Panel*** **i:** Local resolution distribution of final map.

Figure S2. Purification and sequence alignments for $BrxL_C$. ***Panel a:*** Size exclusion chromatography (SEC) chromatogram of BrxL's C-terminal domain ($BrxL_c$). The protein elutes at a volume corresponding to a monodisperse population monomers. ***Panel b***: Structure-based alignment (based on superposition of crystals structures shown in **Figure 1**) of $BrxL_C$ against $RadA_C$ (top panel) and $LonP_C$ (bottom panel). Overall sequence identities for each pairwise alignment are approximately 14%. Neither the previously visualized DNA-contacting residues in $RadA_C$ (blue bold underlined residues in upper alignment) nor the previously established protease catalytic residues in $LonP_C$ (blue bold underlined residues in lower alignment) are conserved in $BrxL_C$.

Figure S3. Size exclusion chromatographic solution behavior of $BrxL_{WT}$ and $BrxL_{E280Q}$, as visualized immediately after initial metal affinity chromatographic purification step. ***Panel a*****:** $BrxL_{WT}$ protein elutes as a mixture of multimers (faster eluting peaks) and monomers (slower eluting peak), each indicated by arrows. To the right is a SDS PAGE gel of the same peak fractions (also indicated with arrows) and neighboring fractions. ***Panel b***: $BrxL_{E280Q}$ displays a larger proportion and more complex elution pattern of multimers and monomers from the same SEC column.

Figure S4. Conformational differences between free versus DNA-bound BrxL subunits. ***Panel a***: Superposition of the NTD+ATPase region within a single BrxL subunit (indicated by bracket; RMSD ~ 0.3 Å) illustrates a corresponding rigid body rotation of the CTD (blue domains) by approximately 5 to 10° (the RMSD values when instead superimposing the CTDs from the free and DNA-bound structures are approximately 0.1 Å). ***Panel b***: Superposition of the same NTD+ATPase region of one BrxL subunit (also

indicated by bracket) showing the conformational differences between free and DNA-bound structures propagated along the entire length of two subunits in the BrxL assemblage associated in a 'tail to tail' arrangement. Subunits from unbound BrxL_{WT} are colored grey; subunits from DNA-bound BrxLE280Q are colored by domain as shown in **Figure 1a**. See also **Supplementary Movies S1-3.**

Figure S5. Assembly of BrxL multimeric complexes requires DNA. ***Panel a***: Purified monomeric BrxL_{E280Q} was incubated alone or with dsDNA (207 bp) and re-run over SEC. In the absence of DNA, BrxL_{E280Q} entirely eluted as a monomer, whereas in the presence of DNA earlier eluting peaks consistent with multimer formation are observed. BrxL_{E280Q} likely purifies bound to ATP that it is not able to hydrolyze. This analysis is consistent with ATP binding to BrxL and the presence of DNA being necessary for multimer formation. ***Panel b***: Purified monomeric BrxL_{WT} was assayed as in panel *a*, with an additional condition in which ATP and DNA were included in the incubation. Under these conditions, earlier eluting peaks (arrow) are only observed when ATP and DNA are present.

Figure S6. Point mutations generated within BrxL protein interfaces. ***Panel a:*** Position of mutations indicated on a ribbon diagram of a single BrxL subunit, bound to dsDNA. Protein is colored by domains as illustrated in **Figure 1a**. ***Panel b:*** Position of same mutations indicated on a ribbon diagram of two BrxL subunits in a tail-to-tail arrangement (again colored by domain), within the context of the larger hexameric ringed protein assemblage (white). ***Panel c***: Position of same mutations along the primary sequence and corresponding domains of BrxL. ***Panel d:*** SDS PAGE of purified mutants. Fractions from the monomeric peak of SEC purification were pooled and concentrated, and 3 µg of each mutant was loaded on the gel.

Supplementary Figure S7. SEC chromatograms of BrxL constructs. All mutants were purified by metal affinity chromatography prior to these SEC runs. Arrows indicate peaks eluting at early retention volumes that likely correspond to multimeric complexes. Fractions from the monomer peak were pooled, concentrated, and used in EMSA and ATPase assays **(Figures 5, 6** and **Supplementary Figure S8**). Several mutants (E79W, R104A, S264A/R265A and L134W (see **Figure 6A**)) have diminished multimeric peaks relative to $\text{BrxL}_{WT,}$ whereas others (E280E, T658W and Q661W) have substantially increased multimeric peaks relative to BrxL_{WT}.

Supplementary Figure S8. EMSA analyses of dsDNA binding by BrxL mutant constructs. All BrxL mutants were analyzed by EMSA analysis with a 207 bp dsDNA probe in the absence or presence of ATP or AMP-PNP, as described in methods and illustrated in **Figure 5** for wild-type and E280Q BrxL**.**

Figure S9. Structural similarity between BrxL and MCM helicase. ***Panel a:*** Superposition of a single BrxL subunit (colored by domains as shown in **Figure 1a**) versus a single subunit from the MCM helicase from the archaeon *Saccharolobus solfataricus* (PDB ID 6MII) (8), demonstrating structural similarity spanning much of the BrxL NTD + ATPase domains (residues 71 to 469), with an approximate backbone rmsd of 4 Å over about 330 residues. ***Panel b***: Superposition of single subunits of BrxL and MCM, with the remaining five subunits of the entire MCM hexameric assemblage shown in grey. The lower panel also shows bound DNA in the central pore of the MCM complex.

Supplementary Figure S10. Structural modeling of BrxC and potential structural similarity to ORC2. BrxC was modeled using the AlphaFold Server. Its N-terminal region is predicted to harbor structural similarity, spanning approximately 267 residues, to a similar region of the origin recognition complex subunit 2 (ORC2) from the archaeon *Aeropyrum pernix* (PDB ID 1W5S).

Supplementary Figure S11. BrxB is predicted to contain a degenerated AAA+ fold with homology to DnaA. The structure of BrxB colored as a spectrum from blue N-terminal to red C-terminal regions) was modeled using the AlphaFold Server. This analysis demonstrated that BrxB adopts a AAA+ fold, which lacks conserved ATP-binding residues in its Walker A and B domains. The DALI server identified DnaA (PDB accession 2ZR4) as a structural homolog, sharing ~4 Å rmsd over 130 amino acids within the AAA+ domain. The top panel shows a structural superposition of the BrxB model with the DnaA structure. The bottom panel shows an alignment between BrxB and DnaA, with secondary structural elemetns of each (and the Walker A and B domains) indicated.

Supplementary Figures

Figure S1a

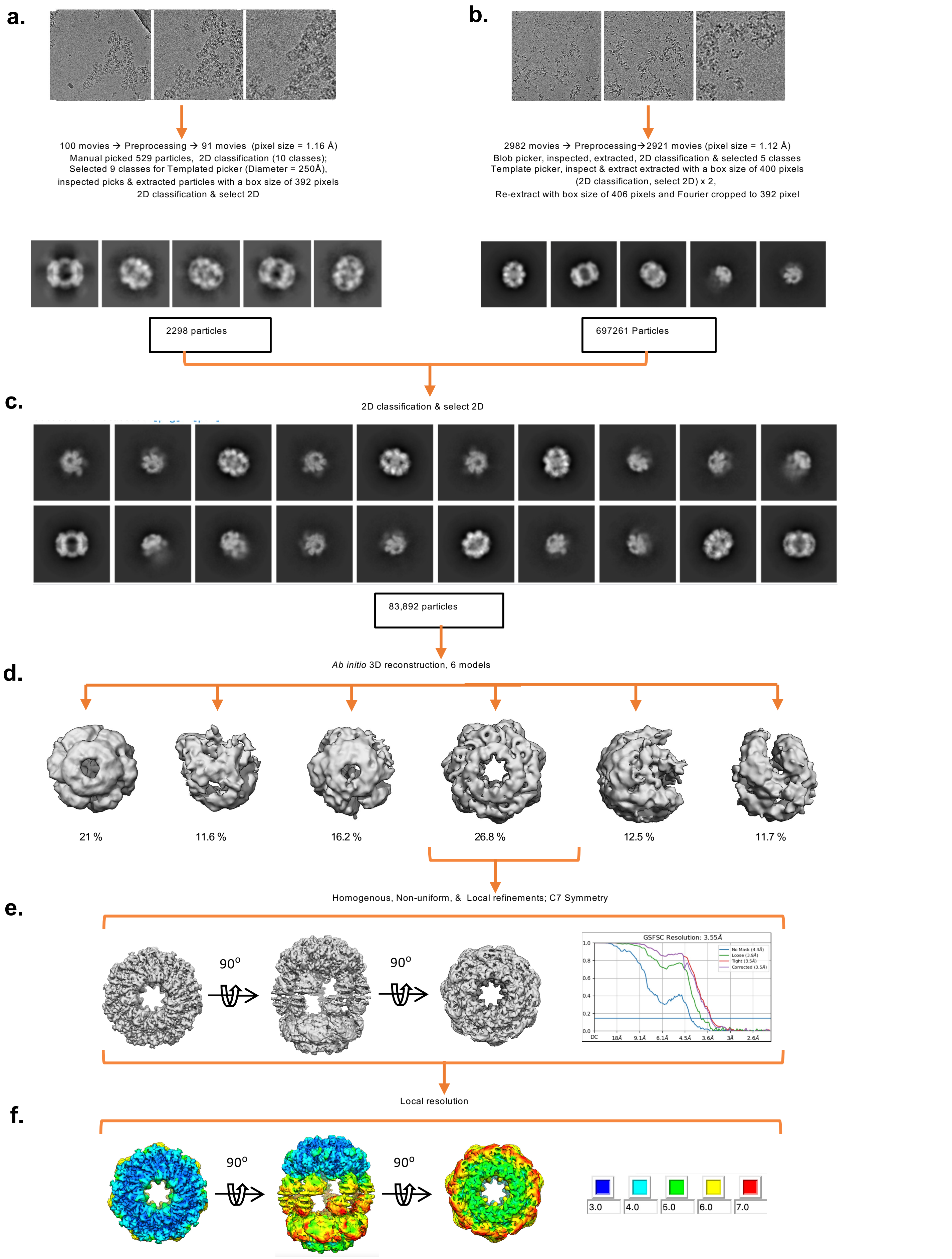
a.
100 movies → Preprocessing → 91 movies (pixel size = 1.16 Å)
Manual picked 529 particles, 2D classification (10 classes);
Selected 9 classes for Templated picker (Diameter = 250Å),
inspected picks & extracted particles with a box size of 392 pixels
2D classification & select 2D
2298 particles
b.
2982 movies → Preprocessing→2921 movies (pixel size = 1.12 Å)
Blob picker, inspected, extracted, 2D classification & selected 5 classes
Template picker, inspect & extract extracted with a box size of 400 pixels
(2D classification, select 2D) x 2,
Re-extract with box size of 406 pixels and Fourier cropped to 392 pixel
697261 Particles
c.
2D classification & select 2D
83,892 particles
d.
Ab initio 3D reconstruction, 6 models
21 %
11.6 %
16.2 %
26.8 %
12.5 %
11.7 %
e.
Homogenous, Non-uniform, & Local refinements; C7 Symmetry
90°
90°
GSFSC Resolution: 3.55Å
f.
Local resolution
90°
90°
3.0
4.0
5.0
6.0
7.0

Figure S1b

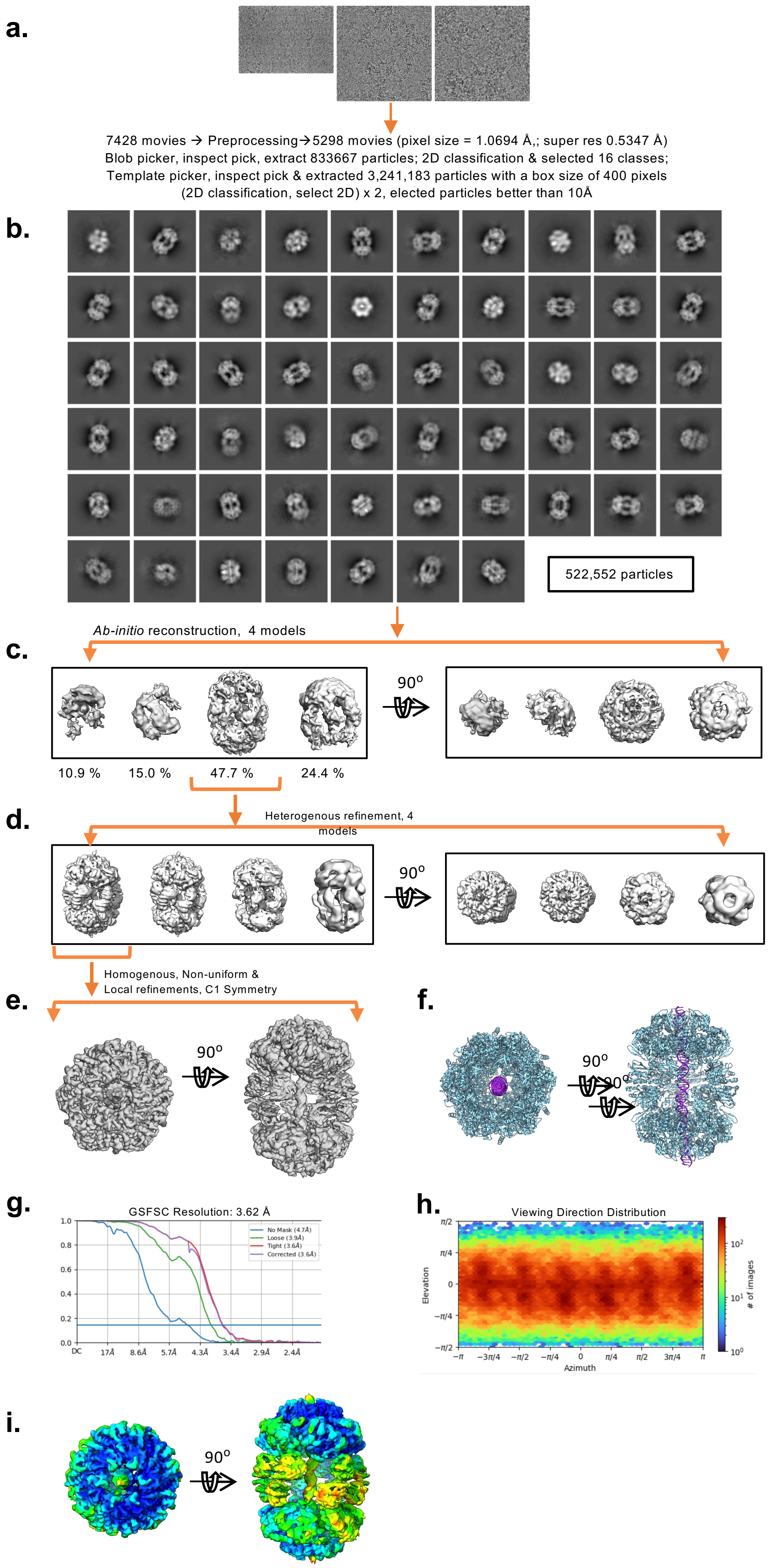

a.
7428 movies → Preprocessing→5298 movies (pixel size = 1.0694 Å,; super res 0.5347 Å)
Blob picker, inspect pick, extract 833667 particles; 2D classification & selected 16 classes;
Template picker, inspect pick & extracted 3,241,183 particles with a box size of 400 pixels
(2D classification, select 2D) x 2, elected particles better than 10Å
b.
522,552 particles
c.
Ab-initio reconstruction, 4 models
90°
10.9 %
15.0 %
47.7 %
24.4 %
d.
Heterogenous refinement, 4 models
90°
Homogenous, Non-uniform &
Local refinements, C1 Symmetry
e.
90°
f.
90°
90°
g.
GSFSC Resolution: 3.62 Å
No Mask (4.7Å)
Loose (3.9Å)
Tight (3.6Å)
Corrected (3.6Å)
1.0
0.8
0.6
0.4
0.2
0.0
DC
17Å
8.6Å
5.7Å
4.3Å
3.4Å
2.9Å
2.4Å
h.
Viewing Direction Distribution
Elevation
Azimuth
# of images
π/2
π/4
0
−π/4
−π/2
−π
−3π/4
−π/2
−π/4
0
π/4
π/2
3π/4
π
10^2
10^1
10^0
i.
90°

Figure S2

a

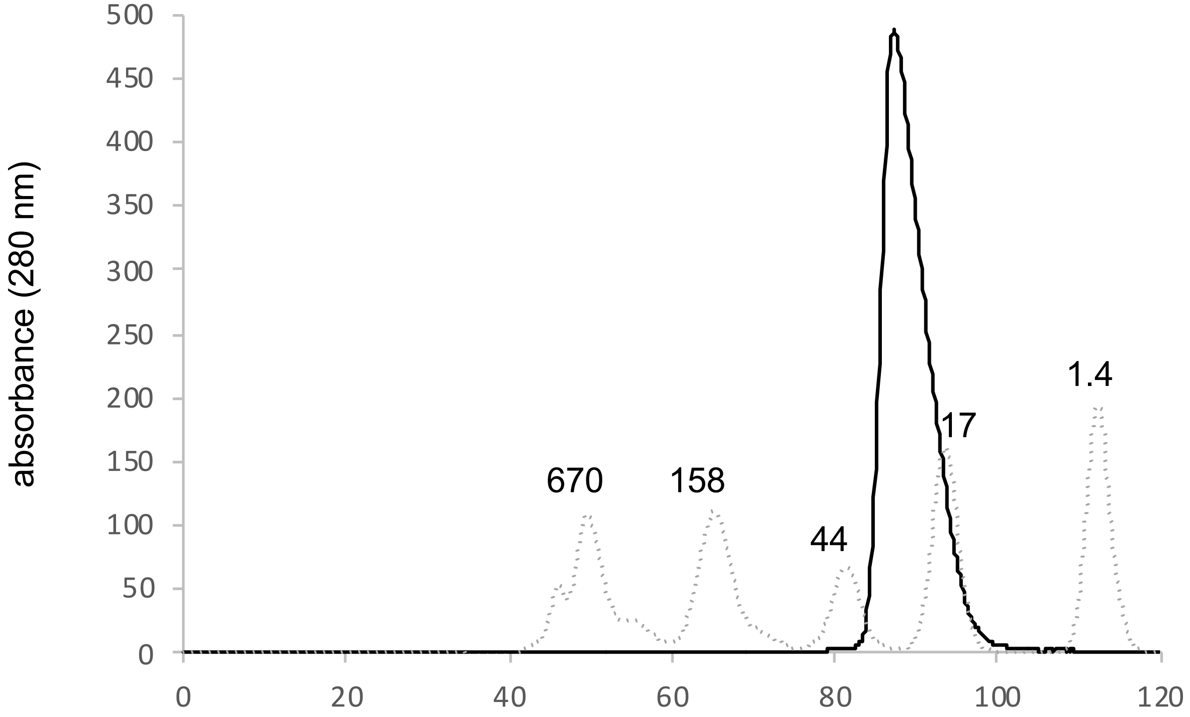

b

($RadA_C$ = 5H45; $LonP_C$ = 1XHK.   LonP Catalytic residues = "X" ; RadA DNA-binding residue = "Y")

```
BrxLC:     498 KPGFLYTIGLSNKGMPGLYRLELQVTKG---SGKLATSGLWNSSSAKEQVKIAFDYFKANASRISGGSKV
                ||      |                |           ||         |
RadAC:     273 VPGSAVALALA-GERALALEVQALAAKTPFPAPRRVVQGL-----DGRRVDVVLAVLERRLG-----LPL

BrxLC:     565 MEHDFHLHVVELQNTGPLSHLALPSLVAFASGLLGRSVQSQMVVLGDMSLGGSVTPVESIAECLQVAFDA
                  |                | |    |  |   ||         |   | | |  |      |     |
RadAC:     332 ANLDVYVNLAGG-LKVQDPGLDLAVALAVYSAVVGRPLPADLALVGEVGLAGEVRRVAGLERRLREGERA

BrxLC:     634 GAKKVALPMSSAADIPTIPVELFTKFQTSFYADPVDAVFKGLG
               |      |                            ||   |
RadAC:     401 GFGRFLHPG--------------------NLKRLQEAVEAYLA
```

(26/181 identities = 14.4%)

```
BrxLC:     498 KPGFLYTIGLSNKGM-PGLYRLELQVTKGSGKLATSGLWNSSSAKEQVKIAFDYFKANASRISG------
               | |  |       |          |                       |       |
LonPC:     458 KVGVIYGLAVLGAGGIGDVTKIIVQILESKNPGTHLLNI----SGDIAKHSITLASALSKKLVAEKKLPL

BrxLC:     561 ---GSKVMEHDFHLHVVELQNTGPLSHLALPSLVAFASGLLGRSVQSQMVVLGDMSLGGSVTPVESIAEC
                                                 |  | ||           |   | | |       |
LonPC:     524 PKKDIDLNNKEIYIQFSQSYSKIDGDSATAAVCLAIISALLDIPLKQDFAITGSLDLSGNVLAIGGVNEK

BrxLC:     627 LQVAFDAGAKKVALPMSSAADIPTIPVELFTKFQTSFYADPVDAVFKGLGVD
                  |   | | |  |     |   |  |                |      |
LonPC:     594 IEAAKRYGFKRVIIPEANMID--VIETE---GIEIIPVKTLDEIVPLVFDLD
```

(26/181 identities = 14.4%)

Figure S3

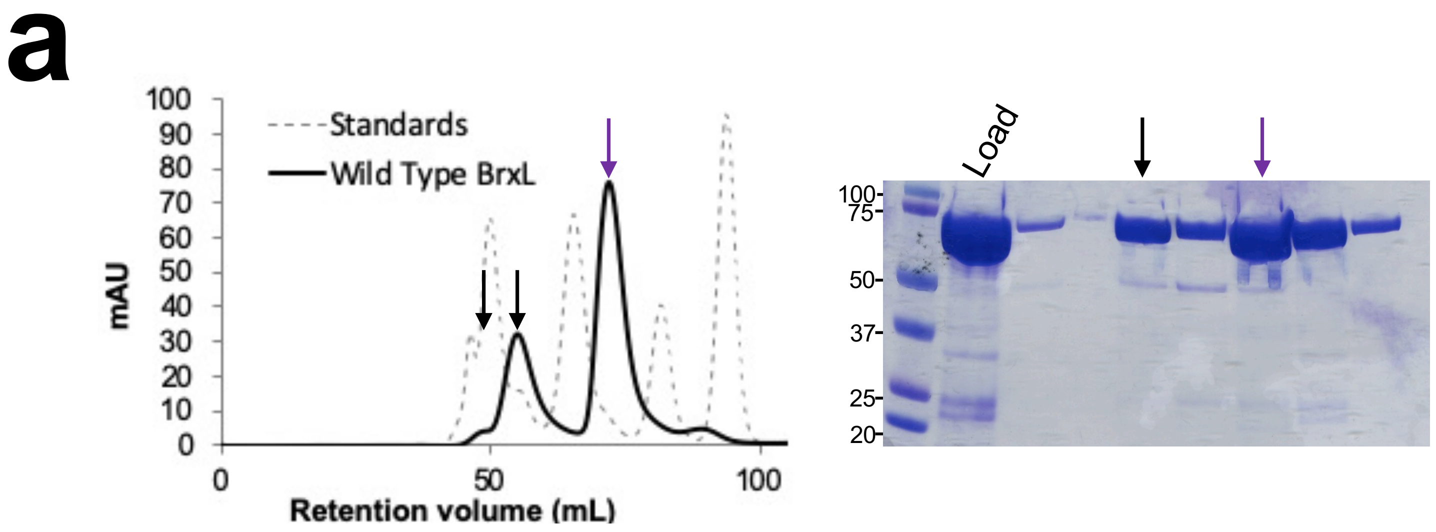
a
100
90
80
70
60
50
40
30
20
10
0
mAU
Standards
Wild Type BrxL
0
50
100
Retention volume (mL)
Load
100
75
50
37
25
20

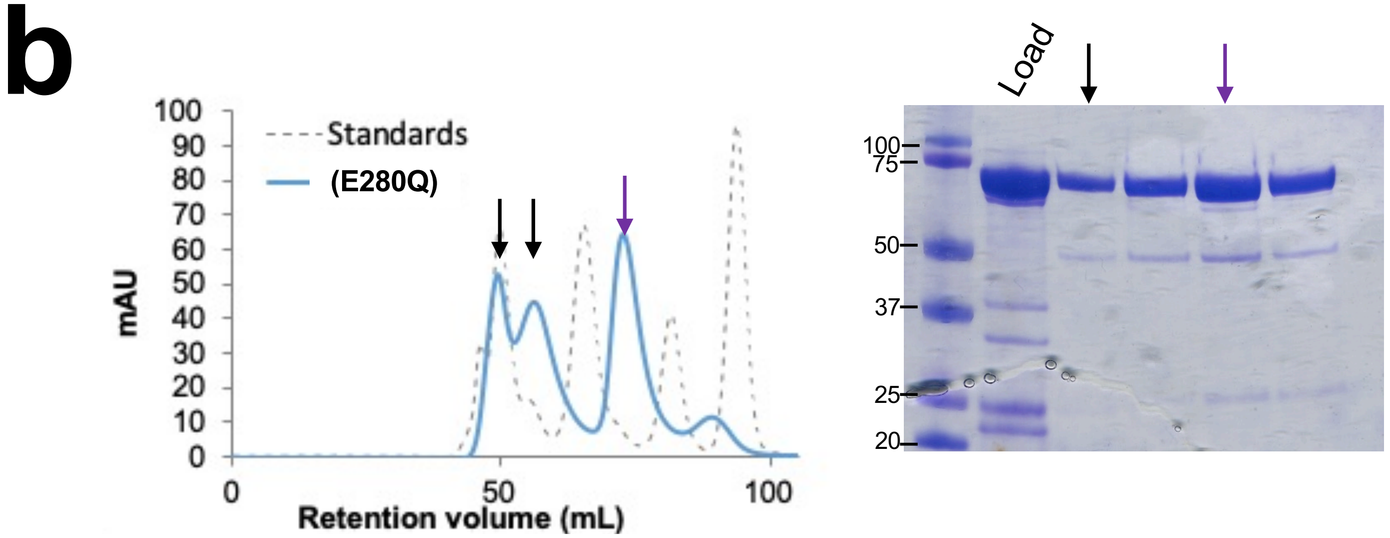
b
100
90
80
70
60
50
40
30
20
10
0
mAU
Standards
(E280Q)
0
50
100
Retention volume (mL)
Load
100
75
50
37
25
20

Figure S4

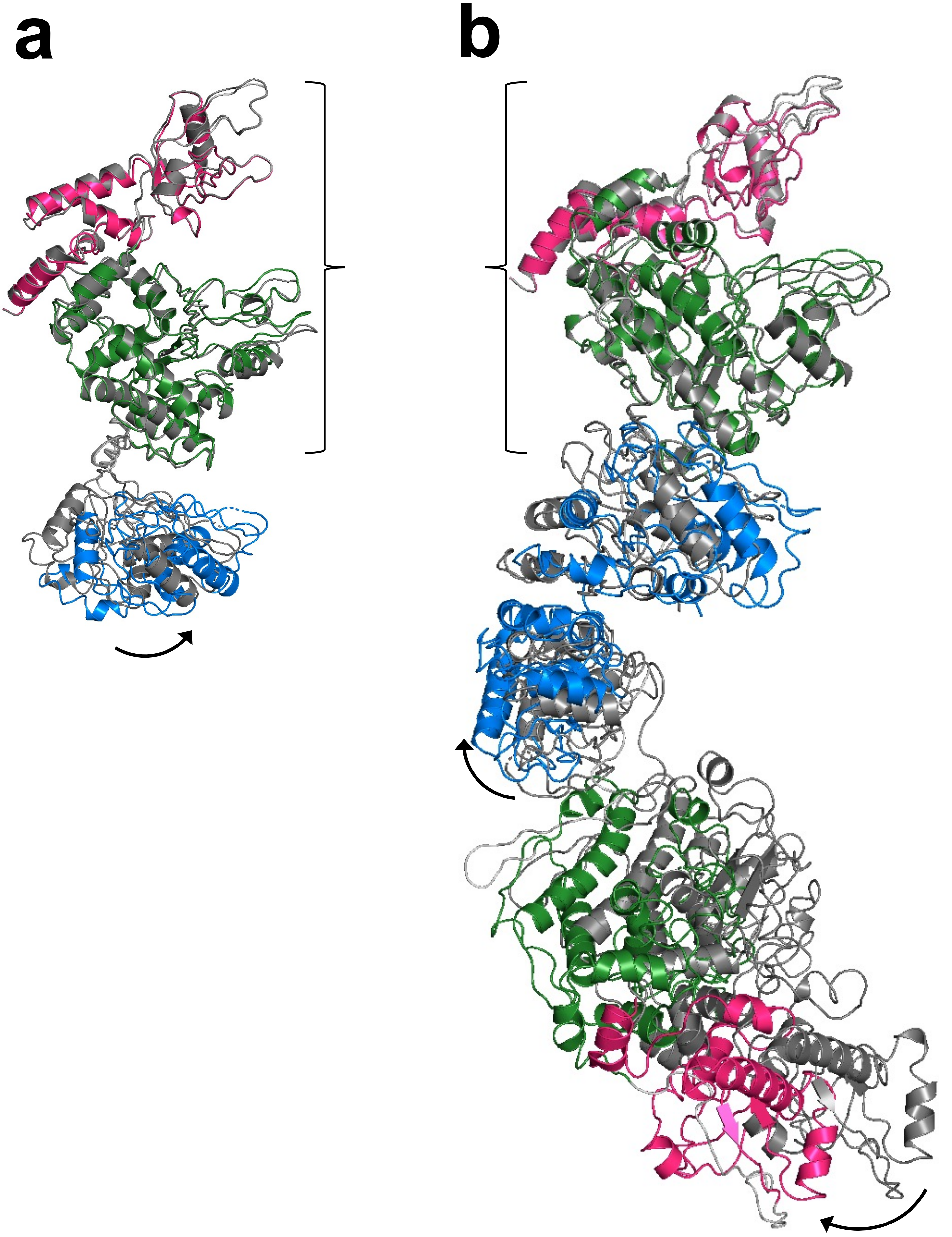

Figure S5

a

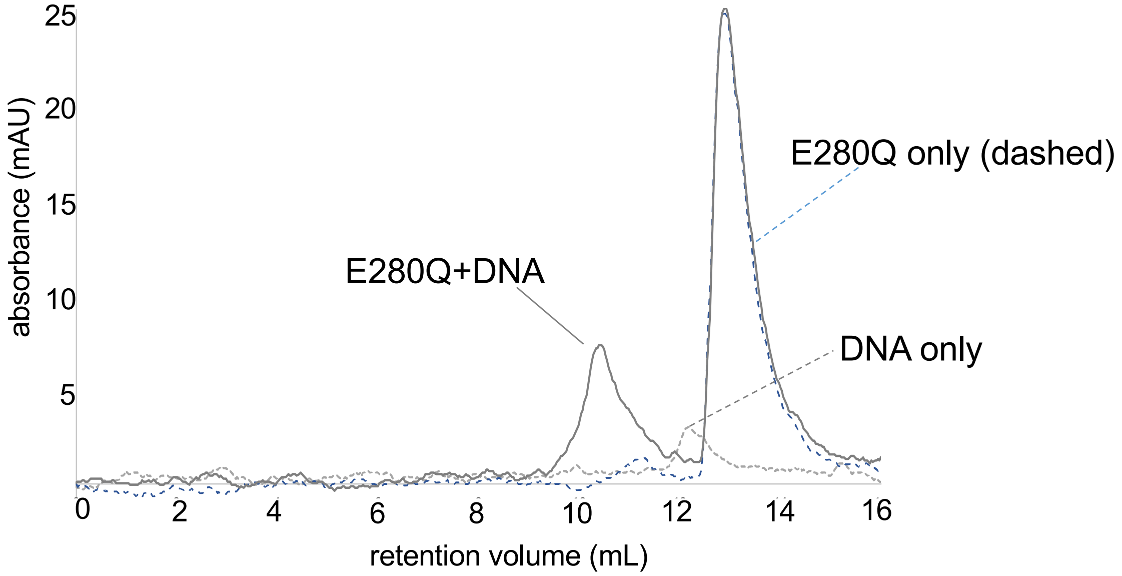

E280Q only (dashed)
E280Q+DNA
DNA only
absorbance (mAU)
retention volume (mL)

b

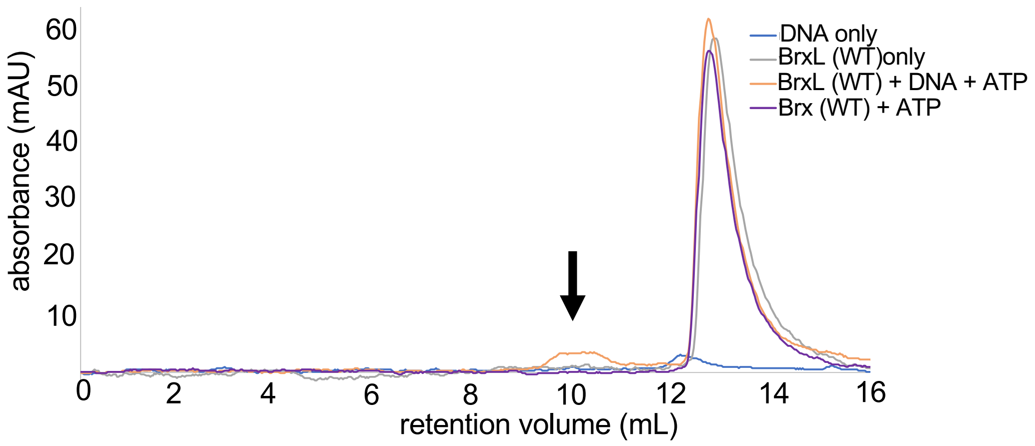

DNA only
BrxL (WT)only
BrxL (WT) + DNA + ATP
Brx (WT) + ATP
absorbance (mAU)
retention volume (mL)

Figure S6

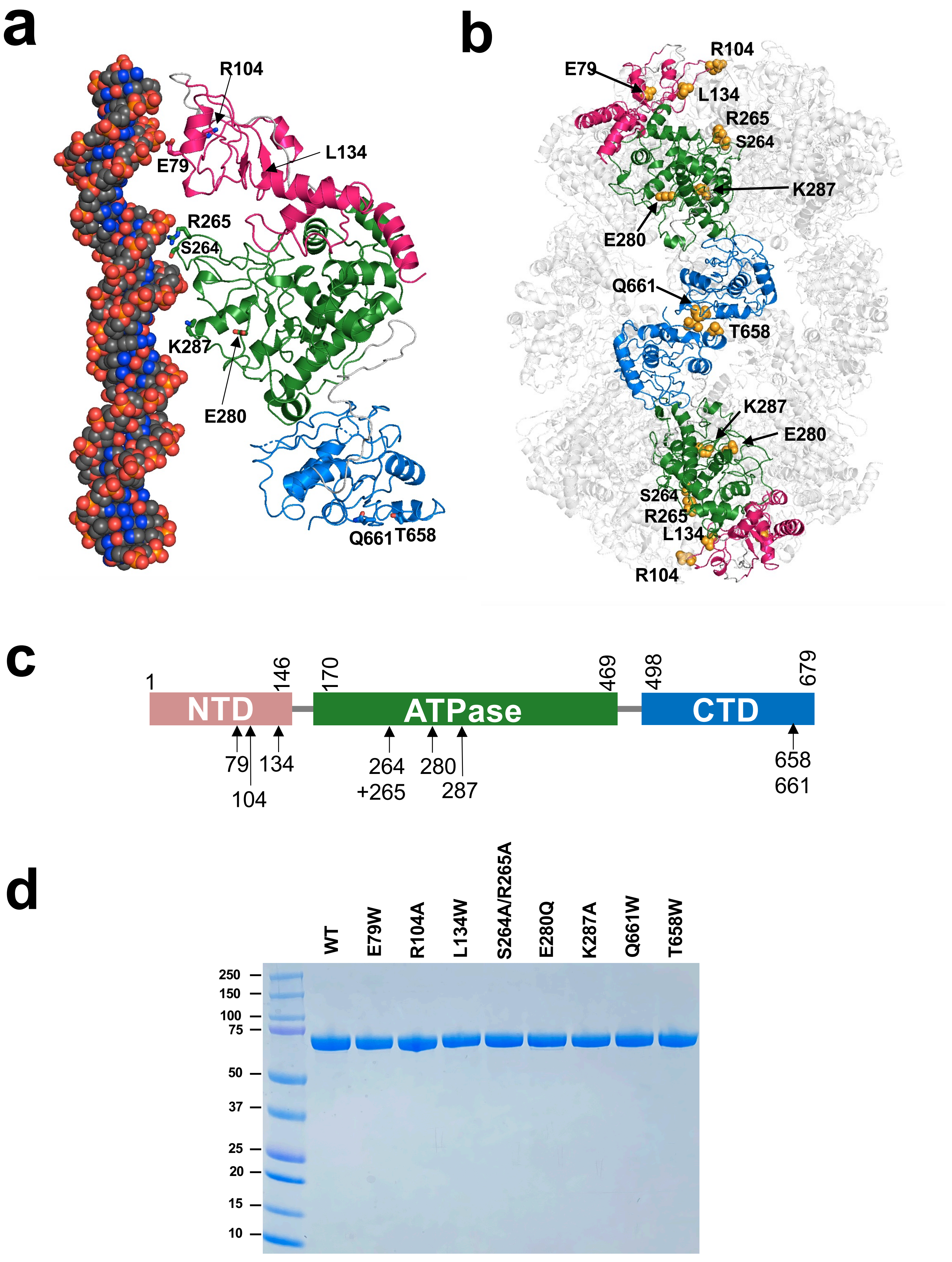

a
R104
L134
E79
R265
S264
K287
E280
Q661
T658
b
R104
E79
L134
R265
S264
K287
E280
Q661
T658
K287
E280
S264
R265
L134
R104
c
1
146
170
469
498
679
NTD
ATPase
CTD
79
134
104
264
+265
280
287
658
661
d
WT
E79W
R104A
L134W
S264A/R265A
E280Q
K287A
Q661W
T658W
250
150
100
75
50
37
25
20
15
10

Figure S7

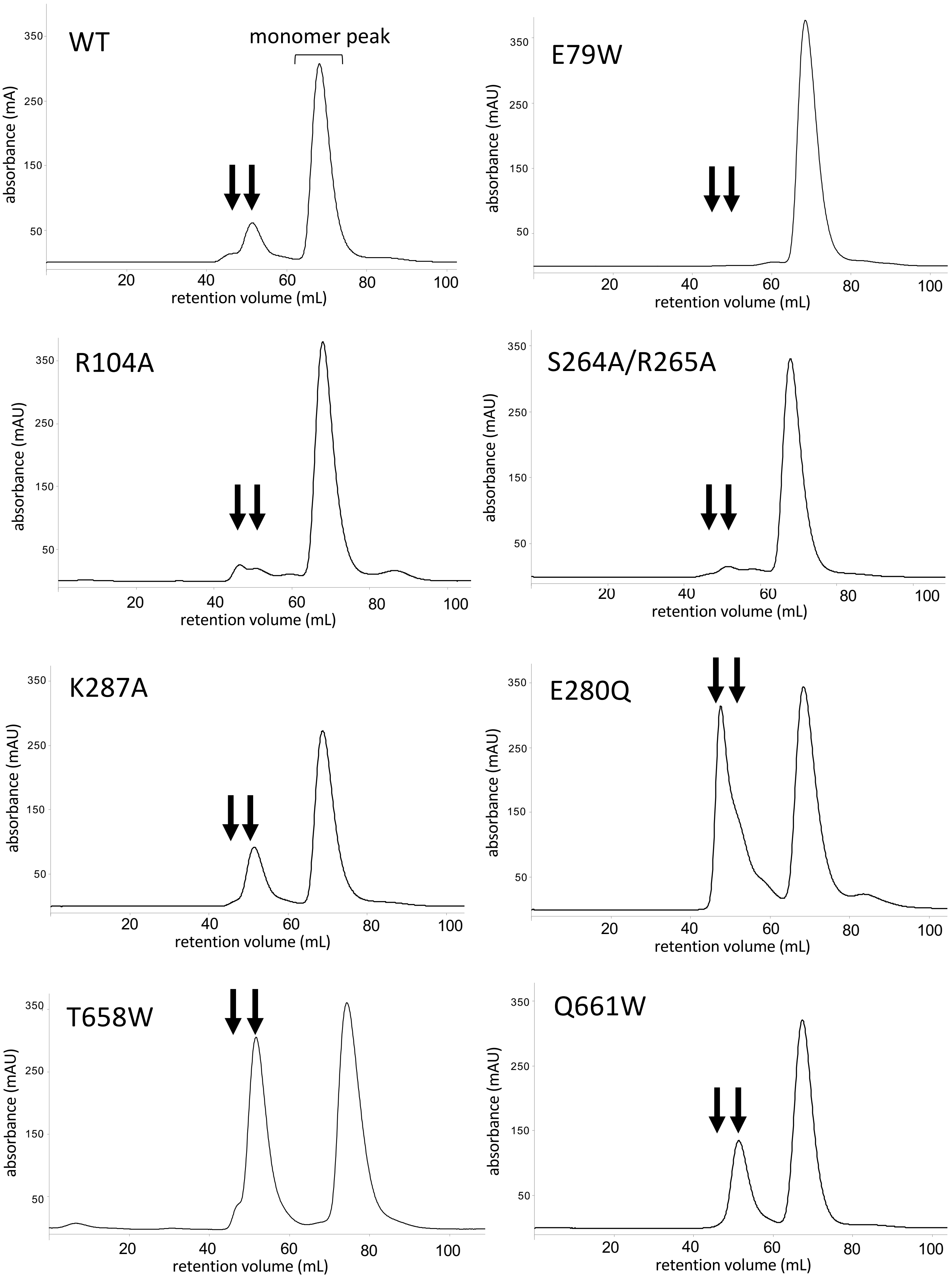
WT
monomer peak
absorbance (mA)
retention volume (mL)
E79W
R104A
S264A/R265A
K287A
E280Q
T658W
Q661W
absorbance (mAU)
350
250
150
50
20
40
60
80
100

Figure S8

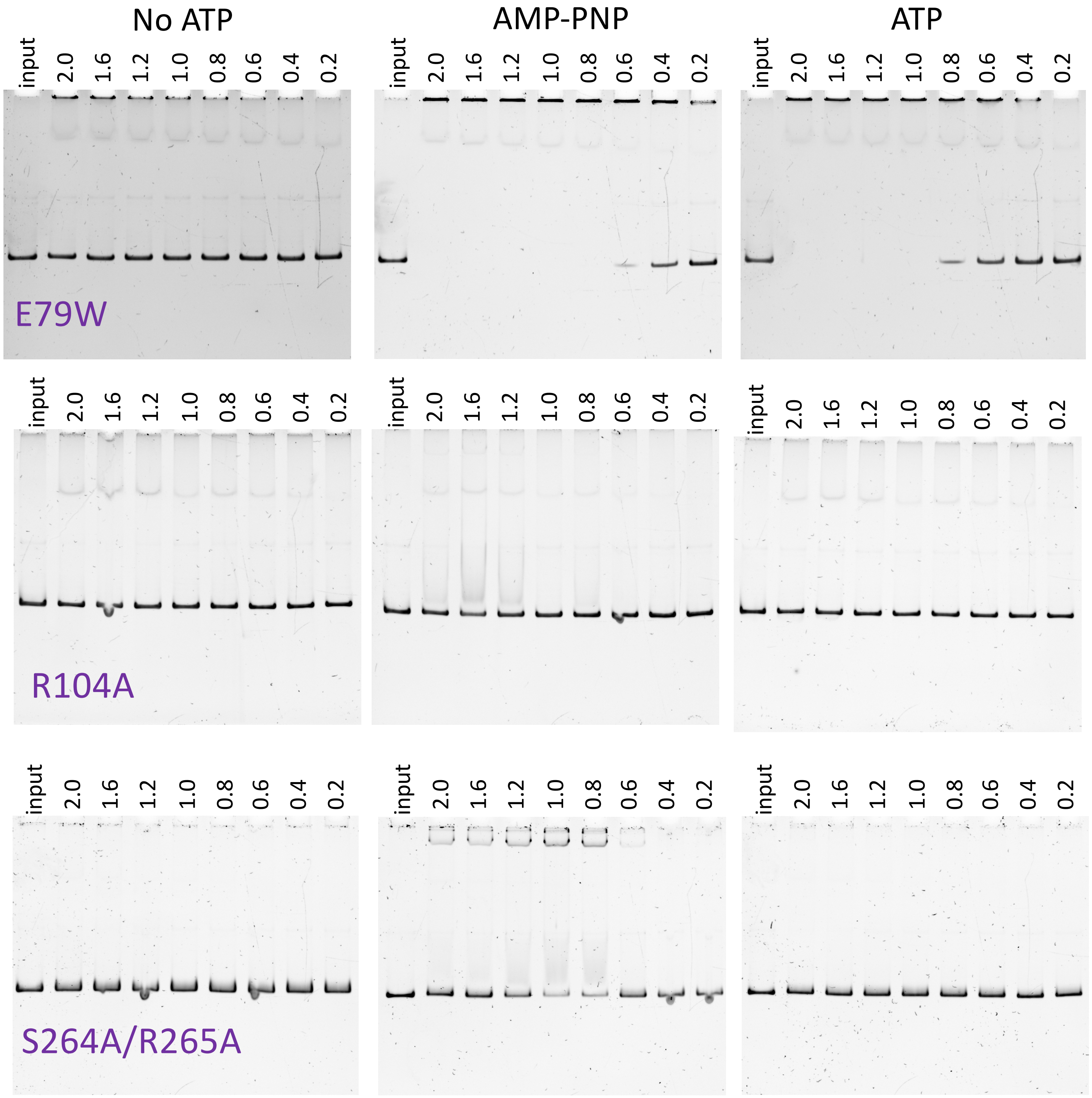
No ATP
AMP-PNP
ATP
input
2.0
1.6
1.2
1.0
0.8
0.6
0.4
0.2
E79W
R104A
S264A/R265A

Figure S8 (cont)

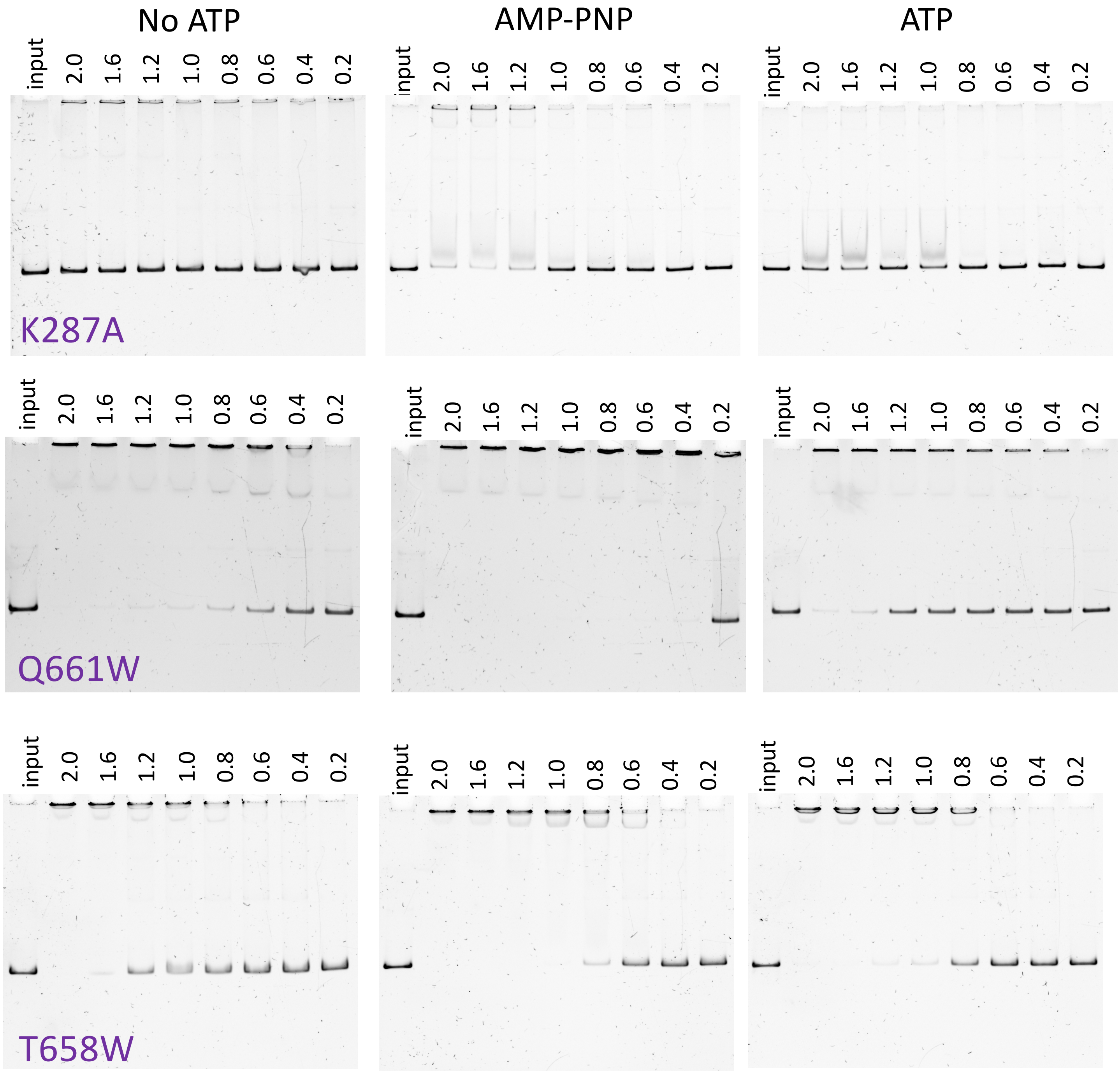

Figure S9

a

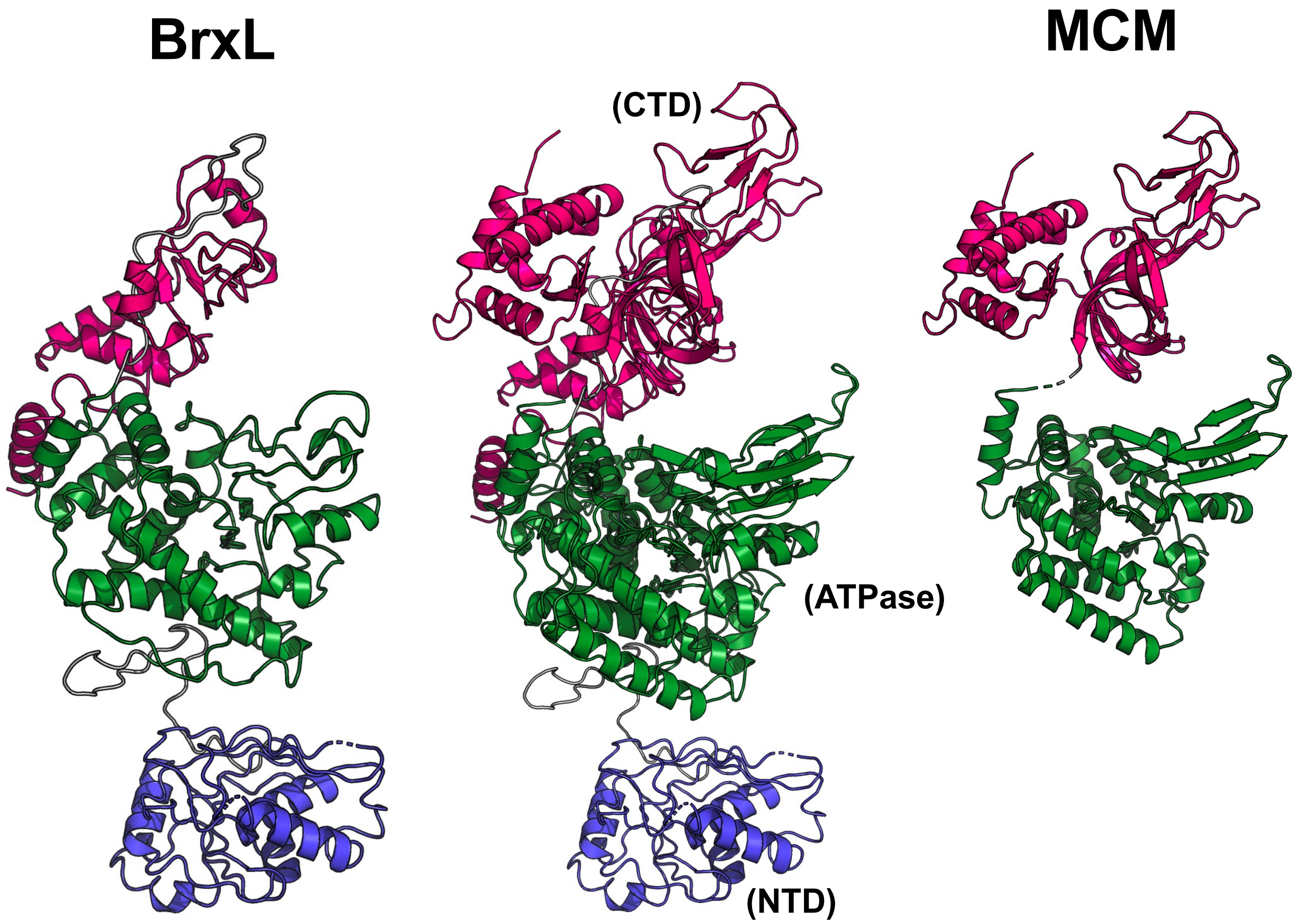

b

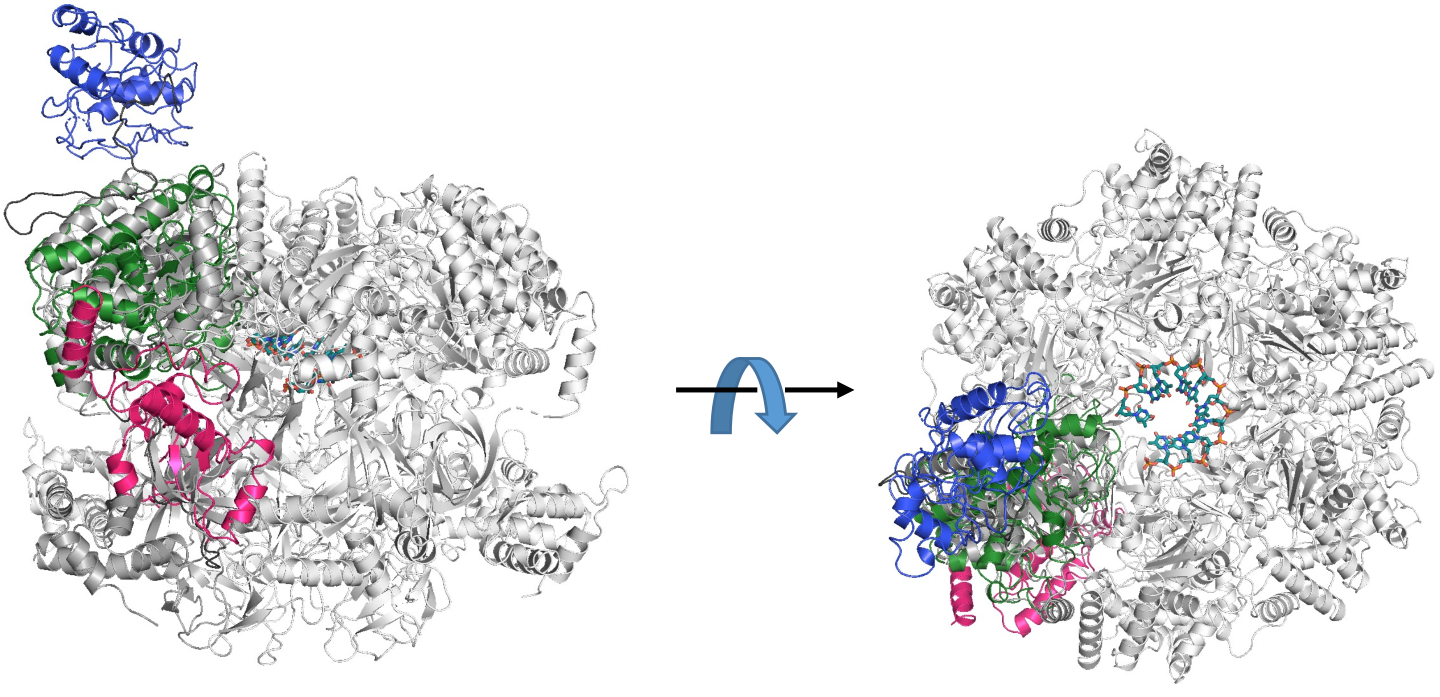

Figure S10

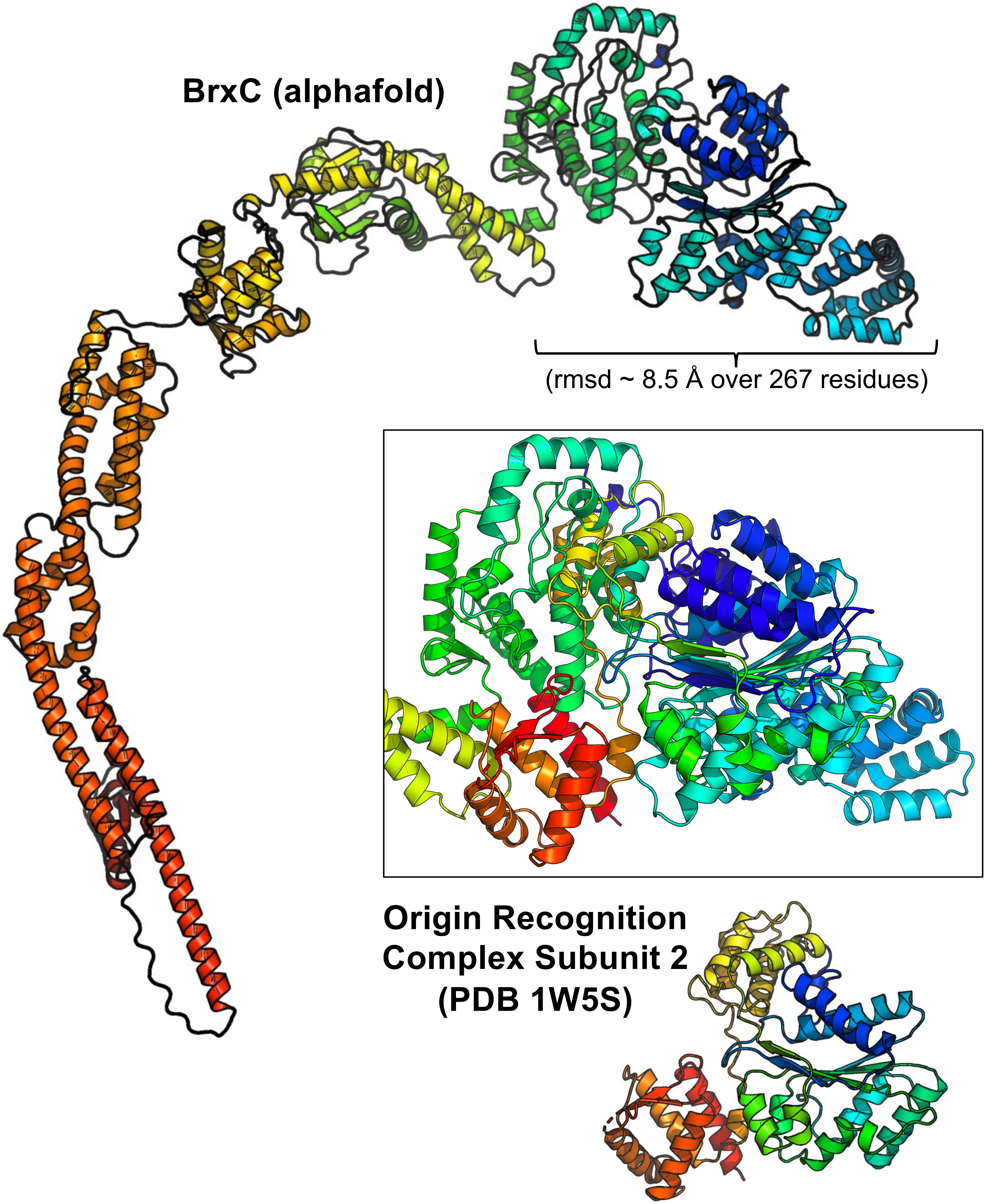
BrxC (alphafold)
(rmsd ~ 8.5 Å over 267 residues)
Origin Recognition
Complex Subunit 2
(PDB 1W5S)

Figure S11

QUERY	Chain	Z	RMSD	lali	nres	%id PDB
BrxB	2zr4-A	7	4	132	240	5

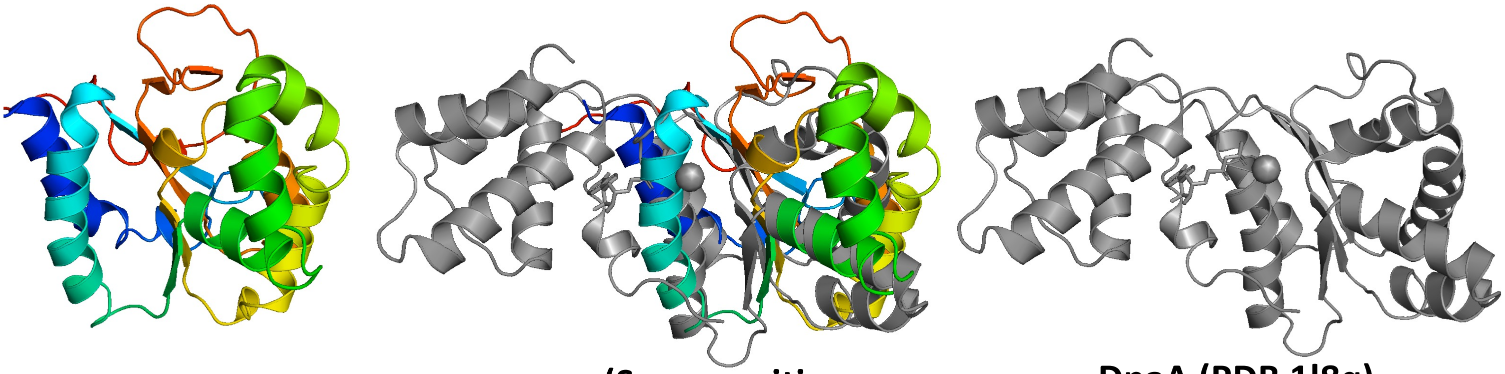

BrxB (Alphafold) **(Superposition** **DnaA (PDB 1l8q)**

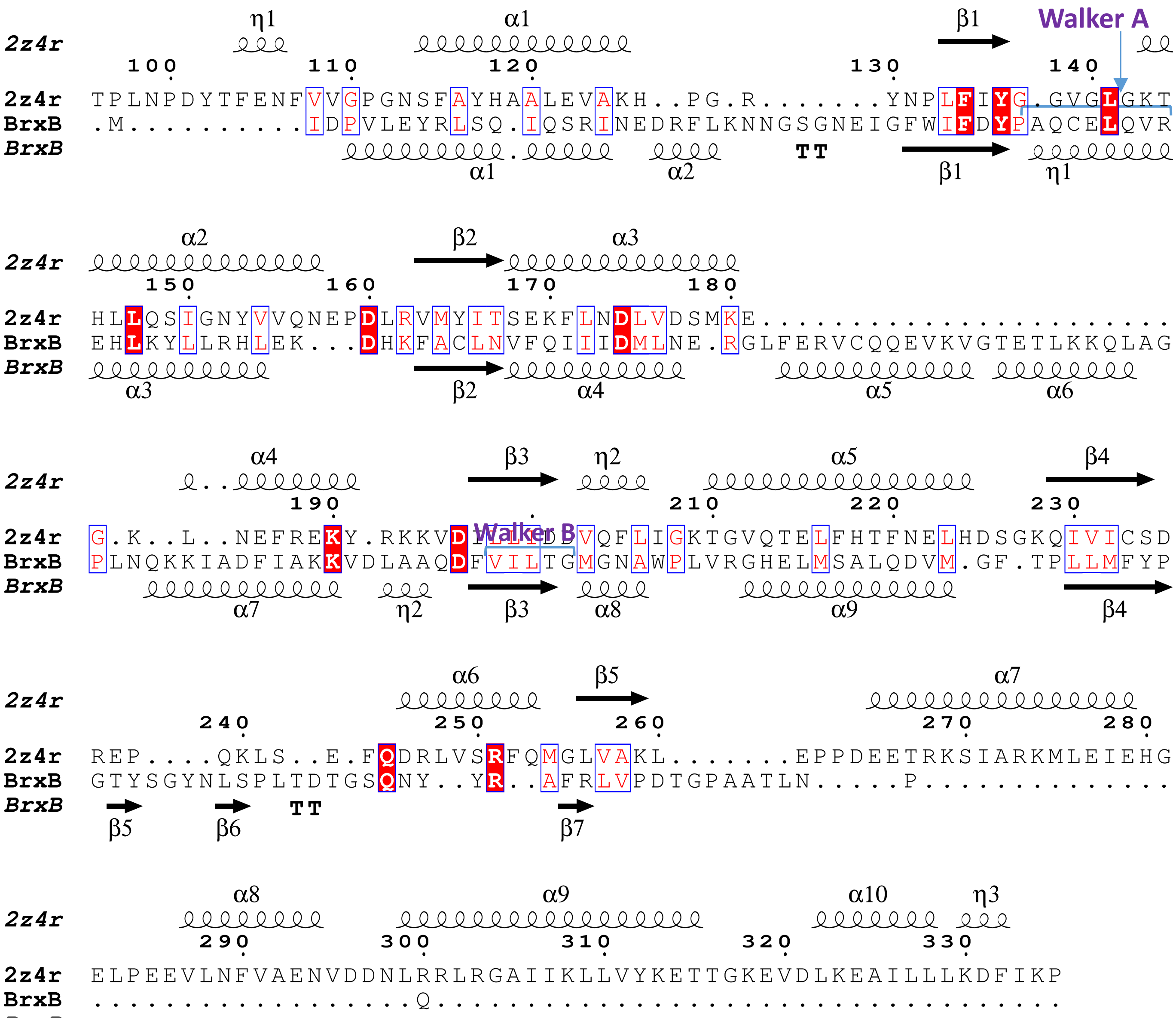

Supplement: gkad083_Supplemental_Files [file gkad083_supplemental_files.zip › Supplementary_Information_Final.pdf]
